# Physiologically-based pharmacokinetic modeling to predict drug-drug interactions of dabigatran etexilate and rivaroxaban in the Chinese older adults

**DOI:** 10.1016/j.ejps.2023.106376

**Published:** 2023-03-01

**Authors:** Jie En Valerie Sia, Xuan Lai, Xinyi Wu, Fan Zhang, Haiyan Li, Cheng Cui, Dongyang Liu

**Affiliations:** a Geriatrics Department, Peking University Third Hospital, Beijing 100191, China; b Drug Clinical Trial Center, Peking University Third Hospital, Beijing 100191, China; c Department of Clinical Pharmacy and Pharmacy Administration, School of Pharmacy, Fudan University, Shanghai 201203, China; d Center of Clinical Medical Research, Institute of Medical Innovation and Research, Peking University Third Hospital, Beijing 100191, China; e Beijing Key Laboratory of Cardiovascular Receptors Research, Peking University Third Hospital, Beijing 100191, China

**Keywords:** PBPK, Dabigatran etexilate, Rivaroxaban, Drug drug interactions, Older adults, AUC, Area Under Curve, BID, Twice daily, BSA, Body surface area, CYP, Cytochrome P450, DAB, Dabigatran, DABE, Dabigatran Etexilate, DAB-G, Dabigatran Glucuronide, DDI, Drug-drug interaction, EHRA, European Heart Rhythm Association, NOACs, Novel Oral Anticoagulants, PBPK, Physiologically-Based Pharmacokinetics, P-gp, P-glycoprotein, QD, Once daily, SD, Single Dose

## Abstract

**Introduction:**

Drug-drug interaction (DDI) is one of the major concerns for the clinical use of NOACs in the older adults considering that coexistence of multiple diseases and comorbidity were common. Current guidelines on the DDI management were established based on clinical studies conducted in healthy adults and mainly focus on the Caucasians, whereas systemic and ethnic differences may lead to distinct management in the Chinese older adults.

**Objectives:**

To investigate the impact of aging on the DDI magnitude between P-gp and/or CYP3A4 inhibitors with dabigatran etexilate and rivaroxaban in older adults, providing additional information for the use in clinical practice.

**Results:**

Compared with the simulated adult, the AUC of the simulated older adults increased by 42–88% (DABE) and 21–60% (rivaroxaban), respectively, during NOACs monotherapy. Simulation on DDIs predicted that verapamil and clarithromycin further increase the exposure of dabigatran by 29–72% and 40–47%, whereas clarithromycin, fluconazole, and ketoconazole increase the exposure of rivaroxaban by 21–30%, 16–24%, and 194–247% in the older adults. Overall, our simulation result demonstrated that aging and DDIs both increased the exposure of NOACs. However, aging does not have a drastic impact on the extent of DDIs. The DDI ratios of young and old older adults were similar to the adults and were also similar between Caucasians and Chinese.

**Discussion:**

We further simulated the interactions under steady-state based on the EHRA guideline (2021). Our simulation results revealed that recommended reduced dosing regimen of dabigatran etexilate during comedication with verapamil and clarithromycin (110 and 75 mg BID for Chinese young and old older adults) will result in exposure (trough concentration) that was either slightly higher or similar to the trough concentration of patients with any bleeding events. Routine monitoring of bleeding risk is encouraged. Further studies on the use of rivaroxaban in Chinese older adults are warranted.

**Conclusion:**

Aging and DDI increases exposure of drug in Chinese older adults. However, aging does not have a drastic impact on the extent of DDIs. Clinical management of DDIs in Chinese older adults in the absence of complex polypharmacy can a priori be similar to the EHRA guideline but routine monitoring of bleeding risk is encouraged when dabigatran etexilate given with verapamil and clarithromycin.

## Highlights

• Exposure of anticoagulants in Chinese older adults is 21–88% greater than in Chinese adults.
• Aging has no drastic impact on drug interaction extent of inhibitors on anticoagulants.
• Absolute increase in drug exposure during drug interaction in older adults is of concern.
• Moderate inhibitors potentially increase bleeding risk of older adults on anticoagulants.
• Reduced dose adaptation results in similar exposure with standard dose monotherapy.

## Graphical abstract

**Figure:**
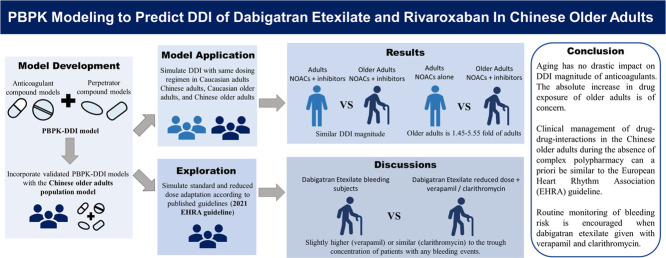

## 1 Introduction

Dabigatran etexilate (DABE) and rivaroxaban are the novel oral anticoagulants (NOACs) approved as the first-line drugs recommended in anticoagulation therapy (Shu et al., 2018; Steffel et al., 2021). While preventing stroke, NOACs also cause up to 3.5% of patients to experience major bleeding annually (Julia and James, 2017). Although older adults with high prevalence make up a very large fraction of clinical population receiving NOACs, they were usually excluded from clinical trials and were underrepresented. Reduced organ functions and multiple medical conditions that became more frequent with older age, and were usually treated with more than one medication, may result in increased risk of bleeding or invalid treatment, increasing the complexity to select a suitable dose during clinical practice (Cavallari and Patti, 2018).

DABE is a prodrug with low absolute bioavailability of 3–7%. It is a substrate of the intestinal P-glycoprotein (P-gp) efflux transporter that is responsible for pumping the compound back into the intestinal lumen. After absorption, DABE is rapidly converted into the active form, dabigatran (DAB), by the plasma esterase, carboxylesterase (CES). The majority of DAB (80–85%) is excreted unchanged in the urine, mainly via glomerular filtration and negligible contributions of tubular secretion or absorption (Knauf et al., 2013). Both dabigatran etexilate and dabigatran are not significantly metabolized by CYP enzymes, but a portion (10–20% of total dabigatran) of DAB is metabolized into dabigatran glucuronide (DAB-G) by UGT2B15 (Blech et al., 2008).

In contrast with DABE, the oral bioavailability of rivaroxaban accounts ≥80%, which is reduced to approximately 60% when the administrated dose is higher than 10 mg and requires food intake to avoid the reduction of oral bioavailability (Stampfuss et al., 2013). Rivaroxaban is also a substrate of P-gp efflux transporter, affecting its absorption and *in vivo* disposition by varying degrees. Two-thirds of the absorbed rivaroxaban is metabolized by hepatic enzyme cytochrome P450 (CYP) into inactive metabolites, primarily by CYP3A4 (main enzyme) and CYP2J2 (minor enzyme), later excreted through urine and feces, while the remaining one-third excreted unchanged in the urine, primarily by P-gp mediated active secretion (BayerPharmaAG, 2014).

P-gp plays an important role in the PK profile of both dabigatran etexilate and rivaroxaban by impairing their intestinal absorption or promoting elimination by the kidney (Gong and Kim, 2013; Wolking et al., 2015). For this reason, potent P-gp inhibitors or inducers are expected to have relevant pharmacological interactions with them increasing or reducing their anticoagulant effect. The P-gp inhibitor, such as verapamil and amiodarone increase the exposure of DAB up to 1.5-fold and clarithromycin, cobicistat, dronedarone, ketoconazole and itraconazole increase the exposure up to 2-fold (Terrier et al., 2021). Moreover, it is generally known that the inhibitors of P-gp and CYP3A4 overlap with each other, co-administrating rivaroxaban with CYP3A4 and/or P-gp inhibitors, such as clarithromycin and verapamil increase the exposure of rivaroxaban up to 1.5-fold, and erythromycin and ketoconazole increases the exposure of rivaroxaban for more than 2-fold (Terrier et al., 2021).

In the 2021 anticoagulation guideline, the European Heart Rate Association (EHRA) had recommended dose reduction in the old older adults (≥80 years old) and also when NOACs were used in combination with inhibitors such as dronedarone, verapamil, erythromycin, clarithromycin, itraconazole, and ketoconazole based on clinical studies and databases (Steffel et al., 2021). However, those recommendations were developed mainly based on younger population and mainly focused on the Caucasians. The management and outcomes for Chinese older adults were uncertain. Ethnic differences and the difference in systemic parameters such as organ function, blood flow, enzyme abundance, and protein level from those of the adults may lead to distinct management of clinical DDIs (Lang et al., 2021; Salem et al., 2013). Compared to the younger patients, the drug-related gastrointestinal bleeding risk of older adults on dabigatran was higher than that of warfarin (Eikelboom et al., 2011). Similarly, the incidence rate of major bleeding events was significantly higher in older adults taking rivaroxaban than in younger subjects (4.63%/100 man-years and 2.74%/100 man-years, *P* < 0.0001) in the ROCKET-AF study (Halperin et al., 2014). In this case, physicians often prescribe doses outside of recommendation (off-label) that are not definitively tested during the registration trials, to avoid bleeding complications.

Physiologically-based pharmacokinetic (PBPK) modeling incorporates the age-related physiological parameters (i.e, body weight/height, organ volumes, blood flows, creatinine clearance), suggested an alternative way to provide an appropriate dosing regimen by conducting virtual clinical scenarios that are not easily or ethically be held on specific populations such as DDIs clinical trials in the older adults (Lau et al., 2020). In a previous study, we developed a Chinese geriatric PBPK population model that can adequately predict the concentration of compounds that are mainly metabolized by CYP1A2, CYP3A4, or eliminated by renal clearance (Cui et al., 2020). By incorporating this population model with the compound models, virtual DDI clinical trials can be simulated to investigate the impact of aging on the DDI magnitude between P-gp and/or CYP3A4 inhibitors with dabigatran etexilate and rivaroxaban in older adults, which have not been investigated in previous clinical trials, to provide additional information for the use of dabigatran etexilate and rivaroxaban in clinical practice.

## 2 Methods

### 2.1 General approach

Generally, we took three steps to investigate the potential impact of advanced aging on the DDI extent of verapamil, clarithromycin, fluconazole and ketoconazole on dabigatran etexilate and rivaroxaban. Supplementary Fig. S1 shows the overall workflow of our study. First, we developed and verified dabigatran etexilate and rivaroxaban compound models. Second, the validated PBPK models were linked to the DDI models and were used to predict the DDIs on dabigatran etexilate and rivaroxaban. Those models were subsequently verified against available clinically observed data. Finally, the verified PBPK-DDI models were leveraged to predict the DDIs of verapamil, clarithromycin, and fluconazole on dabigatran etexilate and rivaroxaban in the older adults.

### 2.2 Development of PBPK model

The construction of PBPK models and the simulation of DDIs were performed using Simcyp Simulator (Version 20.1; Certara, Sheffield, United Kingdom). The dabigatran etexilate and rivaroxaban compound models used were based on published literature, later verified using clinical data (Cheong et al., 2019; Doki et al., 2019; Farhan et al., 2021; Moj et al., 2019; Otsuka et al., 2020).

DAB was administrated in the form of a prodrug, DABE with a standard dose of 150 mg BID or 110 mg BID (reduced dose). The oral absorption of DABE was predicted with the advanced dissolution, absorption, and metabolism (ADAM) model implemented in the Simcyp software. The P-gp-mediated efflux of DABE was described using Michaelis-Menten equation, where the Michaelis constant (K_m_) and maximum flux (J_max_) value was obtained from the reported literature (Doki et al., 2019). A full PBPK model was used to describe the distribution of DABE. DABE is metabolized first into intermediate M1 and M2, then into DAB in the small intestine and liver by CES. Since the sequential metabolism occurs rapidly, only the metabolism of DAB by hepatic CES was considered (Laizure et al., 2014) during model development. 20% of DAB is metabolized to a pharmacologically active metabolite, dabigatran glucuronide acid (DAB-G) when the oral dose of DABE was given (Blech et al., 2008). A metabolite model was developed to predict the plasma concentration. According to the reported literature, the polarity of DAB-G is stronger than that of DAB but has a similar concentration-dependent pharmacodynamic activity, the input parameters of DAB-G were therefore assumed to be similar to that of DAB (Farhan et al., 2021).

The rivaroxaban compound model from Cheong et al. was used without modification (Cheong et al., 2019). Briefly, the ADAM model was used for the prediction of oral absorption, and the parameters related to the intestinal P-gp efflux transporter were obtained from *in vitro* experiments. The intrinsic clearances (CL_int_) of CYP3A4 and CYP2J2 were calculated using a retrograde model with f_m,CYP3A4_ of 0.37 and f_m,cyp2J2_ of 0.29. The effects of renal transporters such as P-gp and OAT3 was also incorporated using the renal mechanistic model in Simcyp software. The detailed input parameters of DABE, DAB, DAB-G and rivaroxaban PBPK compound models were listed in Supplementary Tables S1–S3. Before extrapolating to the scenarios of interest, the predictive performance was first verified against clinically observed data. The details on the inclusion and exclusion criteria of clinical data used in validation of PBPK models can be found in the Supplementary Text S1.

For competitive inhibitors, the inhibition constant (K_i_) for each inhibitor was the parameter required for the development of DDI models. These values were obtained either from the verified perpetrator compound model file provided by the software vendor or from the published literature. The values used were summarized in Supplementary Table S4. DDIs of dabigatran etexilate and rivaroxaban with the inhibitors were simulated. Successful predictions were judged using methods mentioned in the model predictive analysis. Unless otherwise stated, all perpetrator compound models used in this study were obtained from the Simcyp compound model library.

The population models provided in the Simcyp population model library were utilized for the simulations on Caucasian adults (Sim-Healthy Volunteers), Chinese adults (Sim-Chinese Healthy Volunteers), and Caucasian older adults (Sim-Geriatric NEC). For simulations on Chinese older adults, a Chinese geriatric population model that was developed based on the Chinese adult model by considering the aging-related physiological changes such as age distribution, age-height relationship, body surface area, cardiac output and serum creatinine was used (Cui et al., 2020).

#### 2.2.1 Model predictive analysis method

The predictive accuracy of the anticoagulant models was evaluated by comparing the simulated with observed values and visual predictive checks. The observed PK parameters, such as the area under the curve (AUC) and peak concentration (C_max_), were obtained directly from the literature, or from the plasma concentration-time curve using Web Plot Digitizer (version 4.4). The linear trapezoidal method was used to calculate the AUC from time 0 to t, or extrapolated to infinity, while the C_max_ was obtained directly from the plasma concentration-time curve. Arithmetic means or geometric means, whichever available were used. Overall prediction accuracies were deemed successful (i) if the ratio of the simulated to observed PK parameters were within 2-fold of the clinically observed data for the anticoagulant models, or within the acceptance limits calculated using the formula proposed by Guest et al. (Guest et al., 2011) for the DDI models, and (ii) if most of the observed concentration values were within the 5^th^ and 95^th^ percentile of the simulated plasma concentration-time curve (visual predictive check).

### 2.3 Application of PBPK model

The magnitude of DDIs between dabigatran and verapamil (moderate P-gp inhibitor and weak CYP3A4 inhibitor), dabigatran and clarithromycin (moderate P-gp and strong CYP3A4 inhibitor), rivaroxaban and clarithromycin, rivaroxaban and fluconazole (moderate CYP3A4 inhibitor), and rivaroxaban and ketoconazole (strong P-gp and strong CYP3A4 inhibitor) were simulated in the adults (20–59 years old), young older adults (60–74 years old) and old older adults (>75 years old) to assess the impact of aging. The dosing regimens used in the simulations were matched to the published clinical trials conducted in the Caucasian adults, using the validated PBPK-DDI models. In the ICH E7 guideline “Studies in Support of Special Populations: Geriatrics”, it was mentioned that a minimum of 100 patients would usually allow the detection of clinically important differences. Taking into account that in a real-life situation, older adults were usually less easy to be included in the clinical trials, hence the minimum number was used in our simulation. Therefore, each simulation includes 10 trials with 10 subjects in each trial.

## 3 Results

### 3.1 Development of PBPK models

For the rivaroxaban PBPK model, the fraction of metabolism (f_m_) for the contribution of CYP3A4 for hepatic metabolism of rivaroxaban remained unchanged as 0.37, while Otsuka et al. suggested 0.61. The result of our DDI simulation with fluconazole, a CYP3A4 inhibitor that was known to only inhibit CYP3A4 but not CYP2J2 and P-gp transporter, suggested the rationality of the value used (Cheong et al., 2019). The final model parameters and sources of DABE, DAB, DAB-G and rivaroxaban were listed in Supplementary Tables S1–S3, and the input values of the inhibition constant used were summarized in Supplementary Table S4.

### 3.2 Validation of PBPK and PBPK-DDI models

A total of 17 articles related to the human pharmacokinetics of both compounds were collected. The population characteristics and the dosing regimen were listed in Supplementary Tables S5 and S6. The concentration-time profiles and the pharmacokinetic parameters from the 17 clinical studies conducted in Caucasian and Chinese adults and older adults for the single and multiple doses of administrations were compared with the predicted to verify the predictability of developed PBPK model. As a result of visual predictive checks, the plasma concentration-time profile of dabigatran etexilate, dabigatran, dabigatran glucuronide, and rivaroxaban were reproduced well (Supplementary Figs. S2 and S3), and the predicted PK parameters for dabigatran etexilate were comparable to the observed data. The simulated PK parameters for dabigatran etexilate were all within 1.5-fold of the clinically observed data, whereas the predicted PK parameters for rivaroxaban were up to 94% within 1.5-fold, and all within 2.0-fold of the clinically observed data (Supplementary Tables S7 and S8).

**Fig. 1:**
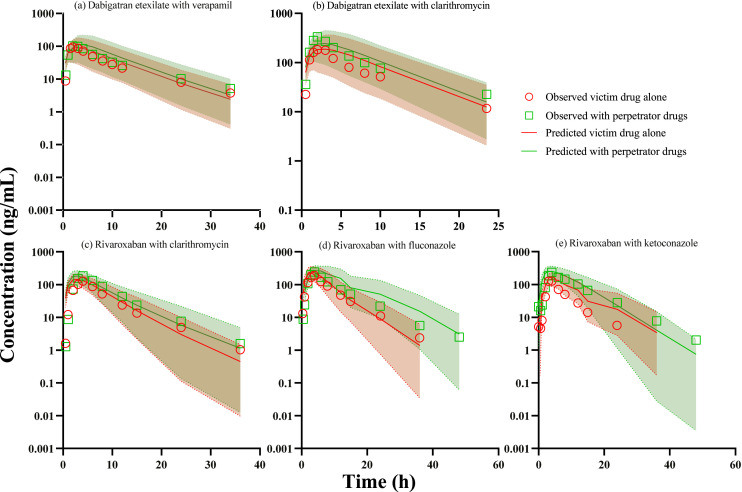
PBPK-DDI model verification of DABE with (a) verapamil, (b) clarithromycin and rivaroxaban with (c) clarithromycin, (d) fluconazole, and (e) ketoconazole in Caucasian adults. The shaded areas show the 5th and 95th percentile of predicted values. The solid lines represent the mean of the predicted value. Markers represent observed values.

Validated compound models were linked together with the inhibitor models to establish PBPK-DDI models. Simulations were performed to validate the predictive performance of developed dabigatran etexilate and rivaroxaban compound model. Simulated PK parameters were up to 92% within 1.25-fold and all within 2.0-fold of the clinically observed data for dabigatran etexilate and rivaroxaban. Furthermore, the predicted extents of inhibition (indicated as DDI ratios) were all within the calculated validation criteria (Guest criterion), as summarized in Supplementary Table S9. The plasma concentration-time profiles were as shown in Fig. 1.

### 3.3 Quantitative simulation to analyze the impact of aging

**Fig. 2:**
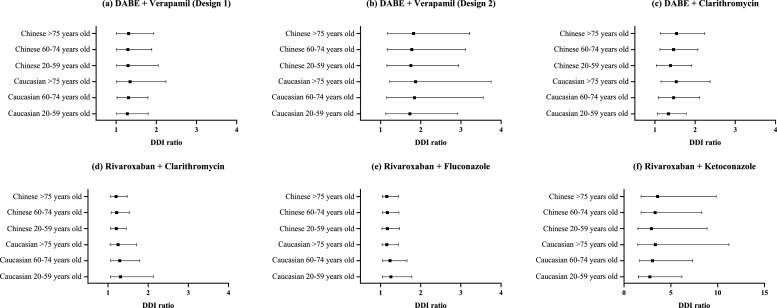
Drug-drug interaction (DDI) of DABE with (a) verapamil (2 h dosing interval), (b) verapamil (concomitantly), (c) clarithromycin, and rivaroxaban with (d) clarithromycin, (e) fluconazole, (f) ketoconazole in Caucasian and Chinese adults (20–59 years old), young older adults (60–74 years old), and old older adults (>75 years old). Black solid dots and lines represent the mean and range of DDI ratio.

**Fig. 3:**
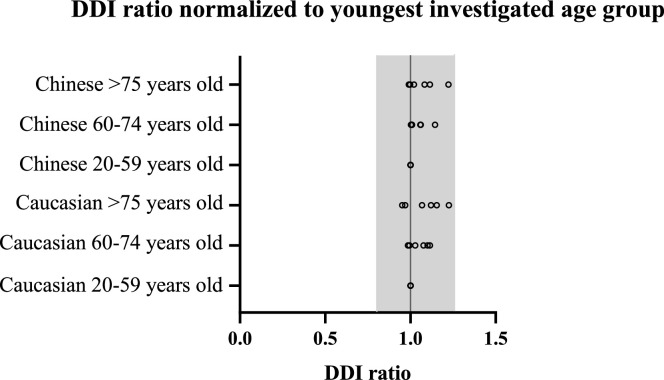
DDI ratio normalized to the youngest investigated age group (20–59 years) for all drugs. Each circle represents a DDI study. The shaded area represents the 1.25-fold interval (bioequivalence criterion).

The fully validated PBPK-DDI models were leveraged to simulate and analyze the impact of aging on the extent of DDI. Clinical studies in adult subjects demonstrated that the DDI ratio of dabigatran etexilate was 1.18 (DDI with verapamil on dosing regimen of 2 h dosing interval), 1.63 (DDI with verapamil given concomitantly) and 1.31 (DDI with clarithromycin), while rivaroxaban given concomitantly with clarithromycin, fluconazole and ketoconazole was 1.52, 1.39 and 2.58 in healthy adults. Under same dosing regimen with the Caucasian adults, the AUC ratio of DDIs in the Caucasian young and old older adults were 1.31-fold and 1.35-fold (Fig. 2(a)), 1.84-fold and 1.87-fold (Fig. 2(b)), and 1.46-fold and 1.53-fold (Fig. 2(c)) for DDIs with dabigatran etexilate, 1.29-fold and 1.25-fold (Fig. 2(d)), 1.24-fold and 1.19-fold (Fig. 2(e)), 3.04-fold and 3.35-fold (Fig. 2(f)), and for DDI with rivaroxaban. Whereas for Chinese adults, and Chinese young and old older adults, the simulated AUC ratio of DDIs were 1.30-fold, 1.30-fold and 1.31-fold (Fig. 2(a)), 1.75-fold, 1.78-fold and 1.82-fold (Fig. 2(b)), and 1.39-fold, 1.46-fold and 1.54-fold (Fig. 2(c)) for DDIs with dabigatran etexilate, 1.21-fold, 1.21-fold and 1.20-fold (Fig. 2(d)), 1.17-fold, 1.17-fold, and 1.16-fold (Fig. 2(e)) and 2.93-fold, 3.32-fold, and 3.58-fold (Fig. 2(f)) for DDIs with rivaroxaban. The DDI magnitudes were similar between the adults and older adults, and were also similar between the Caucasian and the Chinese. A figure of the ratios normalized to the adults were presented in Fig. 3 for better illustration. Our results demonstrated that aging has no drastic impact on the DDI magnitude, regardless of the ethnicity.

**Fig. 4:**
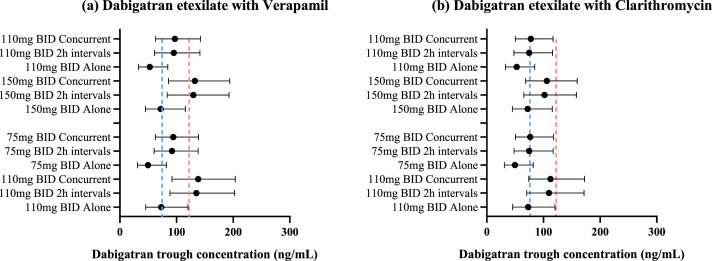
Simulated 10 days steady-state trough concentration of Chinese older adults aged 60–74 years and >75 years for, (a) Dabigatran etexilate with verapamil and (b) Dabigatran etexilate with clarithromycin. Black solid dots were the predicted median values with respective interquartile range. The red dashed line is the observed steady-state median trough concentrations in Chinese patients with major bleeding events (122.1 ng/mL). The blue dashed line is the observed steady-state median trough concentration in Chinese patients with any bleeding events (74.9 ng/mL).

The above simulations were performed using same dosing regimen with the Caucasian adults to evaluate the impact of aging on DDI magnitude. In fact, according to the 2021 EHRA guidelines, for age ≥80 years old, or when dabigatran etexilate was given with verapamil, dose reduction was encouraged. Coadministration of dabigatran etexilate and rivaroxaban with clarithromycin does not require dose reduction but caution should be taken. Caution was also required for coadministration of rivaroxaban with fluconazole but no dose reduction was required unless under complex polypharmacy. Ketoconazole was prohibited from giving together with rivaroxaban. Therefore, we further performed simulation on dabigatran etexilate based on the recommended dose reduction. We simulated dabigatran etexilate given concomitantly with verapamil and clarithromycin for 10 days to reflect the real-world scenario of long-term anticoagulation therapy in the older adults. The results were shown in Fig. 4.

## 4 Discussions

Drug interaction caused by inhibition of P-gp and/or CYP3A4 is one of the most commonly seen mechanisms. To conduct clinical studies without interfering with the ongoing drug therapy in the older adults is certainly challenging due to the comorbidity, poor compliance, ethical factors and risk factors. As a result, dosage optimization became tougher in the absence of guidance from clinical DDI studies, especially in the older adults. There have been reports on quantitative prediction of DDI *in vivo* by constructing various mathematical models (Grillo et al., 2012; Kato et al., 2021; Lingineni et al., 2021). In our study, PBPK modeling and simulation were adopted to predict the DDI in Chinese older adults. PBPK modeling not only takes into account the demographic information and physiological parameters, but also the dynamic changes of inhibitors. Therefore, the PBPK-based DDI prediction model is a more scientific and accurate mathematical model, which can be used to quantitatively predict the *in vivo* pharmacokinetic behavior of inhibitors on enzyme or transporter-mediated disposition.

Before extrapolating to the scenarios of interest, the predictive performance was first verified against clinically observed data using several model predictive analysis methods. In this study, two-fold margin was used to assess the prediction accuracy of dabigatran etexilate and rivaroxaban PK parameters as both dabigatran etexilate and rivaroxaban have pronounced inter-individual variability (Dimatteo et al., 2016; Miklič et al., 2019). To avoid misleading judgments of the assessment on drug-drug interaction by two-fold margin, Guest criterion with 20% of variability was used (Guest et al., 2011). All of the predicted to the observed ratios were within the pre-defined acceptance criteria. The developed models were all able to recapture the observed values.

Based on our simulation results, there was no drastic increase in DDI magnitudes in the older adults under the same dosing regimen compared to the young adults, and the DDI magnitudes were also similar between the Caucasians and the Chinese. Reported studies on the impact of DDI magnitudes in the Caucasians also demonstrated that DDI magnitudes in the older adults were similar to that in the adults (Stader, 2020). A possible explanation for the unchanged magnitudes in our study could be the bioavailability. Rivaroxaban is rapidly absorbed in the gastrointestinal tract. The high concentration of rivaroxaban in the intestinal lumen saturates P-gp, resulting in a less obvious effect on the DDI magnitude. Conversely, dabigatran etexilate which has very low bioavailability and P-gp inhibitors could affect the absorption greatly, but may have already exceeded its maximum tolerable inhibition concentration, hence a less obvious difference in the predicted DDI magnitudes.

Nevertheless, compared with the simulated adult, the AUC of the simulated older adults increased by 42–88% (dabigatran etexilate) and 21–60% (rivaroxaban), respectively, during NOACs monotherapy. Simulation on DDIs predicted that verapamil and clarithromycin further increase the exposure of dabigatran by 29–72% and 40–47%, that is, 1.89 to 3.06-fold and 1.99 to 2.59-fold of that in simulated adults during NOACs monotherapy, while clarithromycin, fluconazole, and ketoconazole increase the exposure of rivaroxaban by 21–30%, 16–24%, and 194–247%, that is, 1.47 to 1.87-fold, 1.45 to 1.80-fold, and 3.81 to 5.55-fold of that in simulated adults during NOACs monotherapy (Table 1). It is necessary to consider the relationship between the increased NOACs concentration and bleeding risk (Schwartz et al., 2021).

**Table 1:** Ratio of AUC under DDI condition normalized to simulated victim drug alone in healthy subjects

Population	Age	Type of data	Dabigatran Etexilate			Rivaroxaban		
			VerI	VerII	Clar	Clar	Flu	Ket
Caucasian	20–59	Predicted	1.31	1.67	1.29	1.32	1.26	2.67
	60–74	Predicted	1.91	2.47	2.01	1.65	1.60	3.81
	75–99	Predicted	2.32	2.95	2.55	1.87	1.78	4.85
Chinese	20–59	Predicted	1.30	1.64	1.34	1.22	1.17	2.82
	60–74	Predicted	1.89	2.32	1.99	1.47	1.45	4.03
	75–99	Predicted	2.46	3.06	2.59	1.83	1.80	5.55

Ver verapamil, Clar clarithromycin, Flu fluconazole, Ket ketoconazole, VerI and VerII is the study on dabigatran etexilate with two hours dosing intervals and given concomitantly, respectively.

A large number of clinical studies have shown that plasma drug concentration of NOACs is correlated to drug efficacy and safety (Foerster et al., 2020; Ruff et al., 2015). Old older adults with significant increased frailty have the greatest risk of bleeding (Šinigoj et al., 2020). The steady-state trough concentration of dabigatran etexilate in Japanese patients > 85 years old was nearly 100% higher (dose-adjusted) than in patients <75 years old (*n* = 25, 0.66 vs 1.20 ng/mL/mg, *P* < 0.05) under similar renal functions (Gommans et al., 2021). In the RE-LY trial where trough concentration was monitored, the increase in drug exposure by 36%, reduce the risk of thromboembolism by 39% with a 16% increased bleeding risk in patients, and when the trough concentration was below 30–50 ng/mL, the effectiveness of dabigatran will be reduced, with an increased risk of thromboembolism (Laureano et al., 2016; Reilly Paul et al., 2014). A study of patients with NVAF also show that steady-state trough concentration (C_trough,ss_) of rivaroxaban is the best exposure indicator for the incidence of major bleeding or clinically relevant non-major bleeding (Zhang et al., 2020). Based partly on these data, it has been suggested that measuring the trough levels would be useful for identifying patients who might benefit from a dose reduction.

Using PBPK modeling, we performed 10 days of steady-state prospective simulations on dabigatran etexilate using the dosing regimen recommended in the EHRA guideline (2021). The predicted steady-state trough concentration (C_trough,ss_) on the ninth day (at post-dose 216 h) of total dabigatran was used as the indicator of the safety (bleeding events, incidence of major bleeding or clinically relevant non-major bleeding), according to the exposure-response (ER) analysis performed in patients with NVAF (Reilly Paul et al., 2014). In a NVAF clinical study on dabigatran etexilate, the observed median dabigatran C_trough,ss_ was 122.1 and 74.9 ng/mL in Chinese patients with major and any bleeding events (Zhu et al., 2022).

In our simulation as shown in Fig. 4(a), the simulated median C_trough,ss_ for Chinese older adults during verapamil co-medicated with standard dose dabigatran etexilate were slightly higher compared to the major bleeding population and were higher than the any bleeding population (132.4 ng/mL (60–74 years old) and 138.1 ng/mL (>75 years old) vs 122.1 ng/mL (major bleeding) and 74.9 ng/mL (any bleeding)). The reduced dose was then simulated. The median C_trough,ss_ reduced, yet higher than the observed C_trough,ss_ of the any bleeding population (97.1 ng/mL (60–74 years old) and 94.2 ng/mL (>75 years old) vs 74.9 ng/mL (any bleeding)) and were also higher than the C_trough,ss_ during standard dose dabigatran etexilate monotherapy in both Chinese young and old older adults. This suggested routine monitoring for bleeding risk. As further dosing reduction could potentially increase the risk of stroke, a change in NOACs used can also be considered in this case.

Whereas for clarithromycin, the simulated median C_trough,ss_ were in between the observed major bleeding population and any bleeding population when clarithromycin was given concomitantly with standard dose dabigatran etexilate (105.9 ng/mL (60–74 years old) and 112.3 ng/mL (>75 years old) vs 122.1 ng/mL (major bleeding) and 74.9 ng/mL(any bleeding)). Unlike verapamil, when reduced doses of dabigatran etexilate were given together with clarithromycin, the simulated C_trough,ss_ were similar to the C_trough,ss_ during standard dose dabigatran etexilate monotherapy, suggesting that a reduced dose should be considered when co-administrating with clarithromycin in the Chinese older adults. However, it should also be noted that the C_trough,ss_ of reduced doses were close to the observed C_trough,ss_ when any bleeding occurred. The results were as shown in Fig. 4(b).

In the clinical drug-drug interaction study conducted by S. Härtter *et al*, two hours dosing interval was shown to be able to minimize the interactions (a lower increase in AUC) during co-medication of dabigatran etexilate with single dose of verapamil (Härtter et al., 2013). Simulations using a dosing regimen with 2 h dosing intervals were performed in our study where multiple doses of verapamil was given concurrently with multiple doses of dabigatran etexilate. Although 2 h dosing intervals were able to reduce the increase in AUC, our simulation results suggested that this dosing regimen has lesser effect on lowering the increase in trough concentration. The simulated trough concentration of dabigatran under dosing regimen of 2 h dosing intervals was similar to that when multiple doses of inhibitors and dabigatran etexilate were given concurrently. The accumulation of verapamil under repeated dosing (Schwartz et al., 1985) and the prolonged verapamil elimination advanced aging (Schwartz, 1990) could possibly explain the phenomenon of lessening effect.

Due to the lack of available bleeding concentration data in rivaroxaban, only evaluation on dabigatran etexilate was performed. These simulation results guided us to propose the following dosage regimens individualized for: (i) Chinese young older adults, 110 mg BID dabigatran etexilate with routine monitoring; and (ii) Chinese old older adults, 75 mg BID dabigatran etexilate with routine monitoring, or change in medication, when giving verapamil or clarithromycin with dabigatran etexilate in long term anticoagulation treatment.

Although routine measurements of NOACs concentration during clinical use were neither recommended nor included in the treatment guidelines, it was still encouraged in the Chinese patients as research has shown that the bleeding rate appears to be higher in Asians compared to the non-Asians (Lip et al., 2015). Routine monitoring was also encouraged in patients with conditions such as polypharmacy, as it might alter the absorption of NOACs or when receiving NOACs doses outside published guidelines, to evaluate potential bleeding risk based on elevated exposure (Sukumar et al., 2021; Sukumar et al., 2019). Our results provide exposure information (indicated as trough concentration) as a reference for clinical NOACs dosing regimen decision-making.

Several limitations should be acknowledged. First, this study only involves the competitive and reversible inhibitors. Concentration-dependent or time-dependent inhibition should not be viewed the same way as it alters the concentration differently (Deodhar et al., 2020).

Second, the regulation or expression of metabolic enzymes and transporters might be affected by advancing age (Stader et al., 2021). The increase in age may decrease the ability of intestinal P-gp to mediate the efflux of the drug, increasing the drug-related safety risks. P-gp is the glycoprotein encoded by Multidrug Resistance 1 (MDR1), which is highly expressed in intestinal tissues and is one of the imperative factors affecting the bioavailability of many substrates (Elmeliegy et al., 2020). An early small sample of clinical research on Japanese older adults aged 67–85 years had found that the intestinal expression of MDR1 reduced by about 60% (*n* = 8, *P* > 0.05) compared to the adults (Miki et al., 2005). A more recent proteomics study on 24 months old aged mice also found that the expression of multiple intestinal transporters that are in the same family as P-gp was down-regulated by about 50%, similar to that of humans. Similarly, the abundance of highly expressed CYP3A enzymes that are responsible for mediating phase I metabolism and drug-drug interactions of clinical commonly used drugs were also affected by aging. The CYP3A activity and abundance of liver microsomes decreased by 49% and 67%, respectively in aged mice (*n* = 12, 24–25 months old, equivalent to human aged 75–80 years) when compared to younger mice (*n* = 10, 2–3 months old, equivalent to human aged 16–22 years). A clinical study extracting liver tissues also found that age is negatively correlated to CYP3A abundance. For every 10 years increase in age, the CYP3A abundance will decrease about 8% (George et al., 1995). However, the change of enzyme CYP3A and P-gp transporter abundance advancing age is still controversial and data are currently sparse in the Chinese older adults. Hence, they were assumed to be the same as in Chinese adults in our PBPK model.

Parameter sensitivity analysis performed based on the consideration of multiple factors such as age, height, sex, weight and renal function on dabigatran etexilate PBPK model revealed that the expression of intestinal P-gp transporter is the imperative factor as it mediated the change of drug exposure up to roughly 2 times. Whereas for rivaroxaban, sensitivity analysis performed demonstrated that both intestinal P-gp abundance and CYP3A4 were sensitive parameters but were less pronounced than dabigatran etexilate and renal P-gp was an insensitive parameter (Supplementary Fig. S4). We perform a simulation by considering 8%/10 years decrease in CYP3A4 abundance (George et al., 1995) and 25.5% decrease in P-gp abundance (Cui et al., 2022), noticed that the change in exposure of DAB and rivaroxaban was less than 20%. Therefore, the potential impact of the assumption of Chinese older adults having the same P-gp and CYP3A abundance as the adults may not have a significant effect on the DAB and rivaroxaban. Further exploration of the effect of reduced P-gp and CYP3A abundance and function on the DDI magnitude shall be conducted in our future studies.

Moreover, our current PBPK model considered the aging-related changes in demographic information, cardiac output, organ weights, and glomerular filtration rate (GFR). The disease-specific pathophysiological change in GFR have yet to be accounted. The simulated older adults with lower GFR shows a greater exposure change (higher C_trough,ss_) during DDI (data not shown), suggesting that varying degree of kidney function loss can lead to a more significant increase in the risk of bleeding. Therefore, the reduced regimen proposed in our study should be viewed with caution in Chinese young and old older adults with renal impairment (Cheong et al., 2019). Further clinical studies in the older adults, especially in the old older adults are warranted to support the model-based hypothesis in our study.

## 5 Conclusion

Older adults are generally more vulnerable to the adverse effect of anticoagulants. Our study highlighted the increase in the plasma exposure of anticoagulants in the older adults when given the same dosing regimen as the adults. Although the predicted age-related changes of DDI magnitude were similar between the adults and the older adults, the simulation on inhibitors with dabigatran etexilate and rivaroxaban predicted that the AUC ratio is 1.45-fold to 5.55-fold of that in simulated adults on NOACs monotherapy. Further evaluation on the suitability of dose reduction recommended based on the latest EHRA guideline revealed that the clinical management of DDIs in Chinese older adults in the absence of complex polypharmacy can a priori be similar to the EHRA guideline but routine monitoring of bleeding risk is required when dabigatran etexilate co-administrated with verapamil and clarithromycin. As usage follows prevalence, FDA encourages the clinical research agencies to enroll clinical subjects based on the proportion of older adults in the population with the treatment indication. We prospectively simulated concentration data under DDI conditions, which can provide reference data for future clinical studies or as a reference for clinical NOACs dosing regimen decision-making.

## CRediT authorship contribution statement

**Jie En Valerie Sia:** Methodology, Investigation, Writing – original draft, Visualization. **Xuan Lai:** Investigation, Visualization. **Xinyi Wu:** Writing – review & editing. **Fan Zhang:** Writing – review & editing. **Haiyan Li:** Writing – review & editing. **Cheng Cui:** Conceptualization, Methodology, Writing – review & editing. **Dongyang Liu:** Conceptualization.

## Declaration of Competing Interest

Valerie Sia Jie En, Xuan Lai, Xinyi Wu, Fan Zhang, Haiyan Li, Cheng Cui and Dongyang Liu declare that they have no potential conflicts of interest that might be relevant to the contents of this manuscript.

## Data Availability

Data will be made available on request.

## Appendix Supplementary materials

Image, image 1

Image, application 2

Image, application 3

Image, application 4

Image, application 5
