# Supplementary figures and images for: Physiologically-based pharmacokinetic modeling to predict drug-drug interactions of dabigatran etexilate and rivaroxaban in the Chinese older adults
Source: Eur J Pharm Sci. 2023 Mar 1;182:106376. doi: 10.1016/j.ejps.2023.106376 (PMC9883662; doi:10.1016/j.ejps.2023.106376)

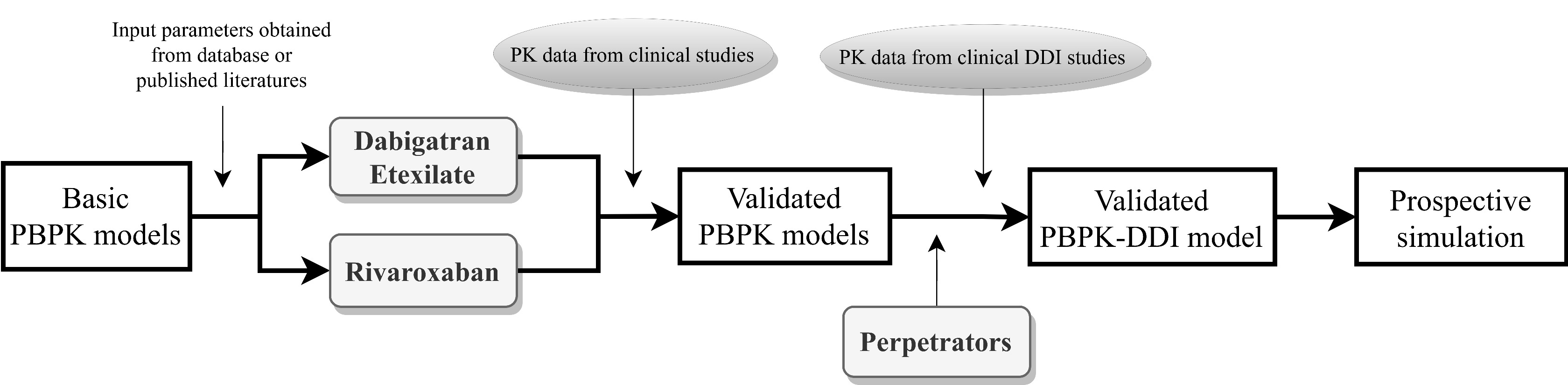

Supplement: Supplementary file 1 [file mmc1.jpg]

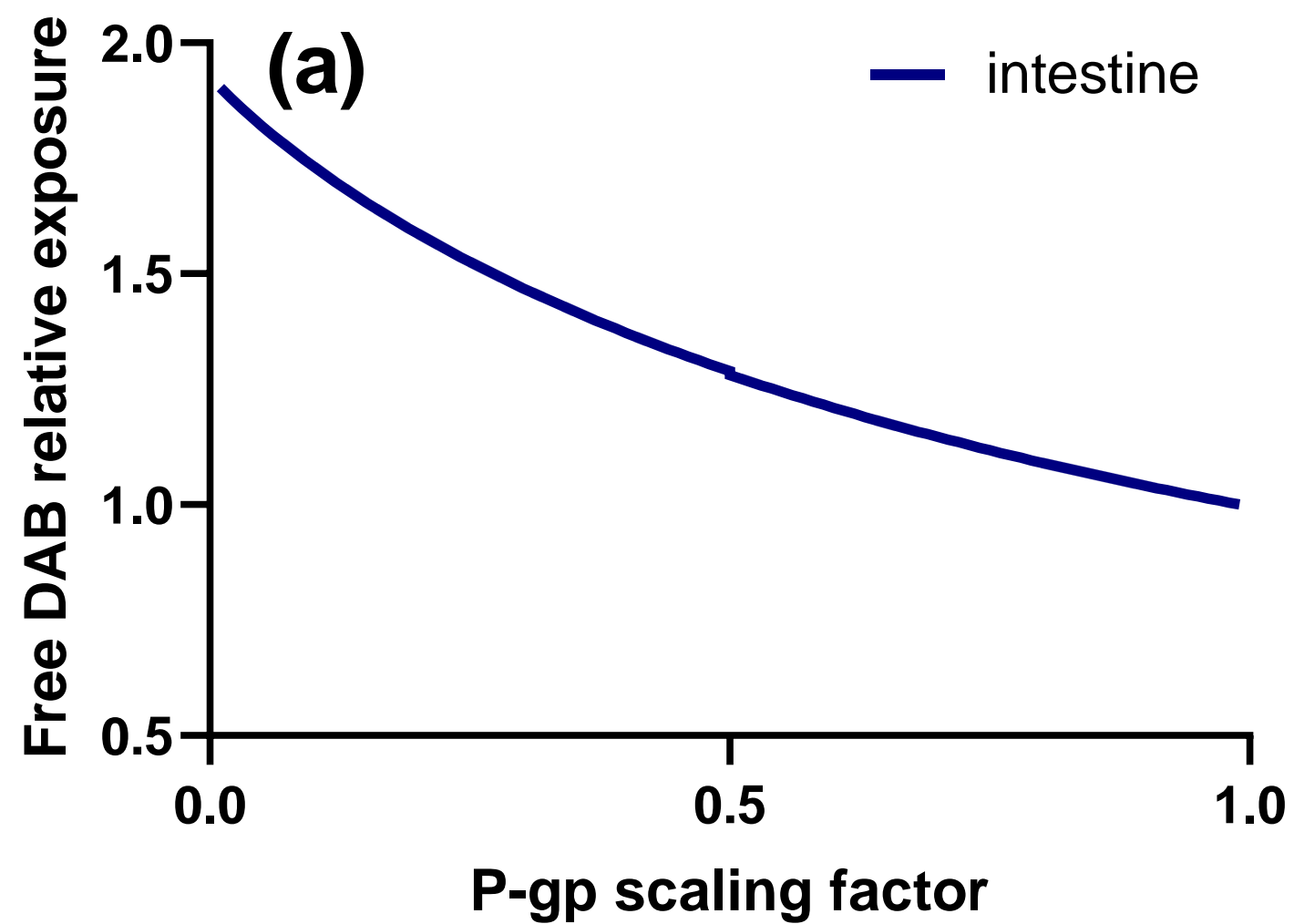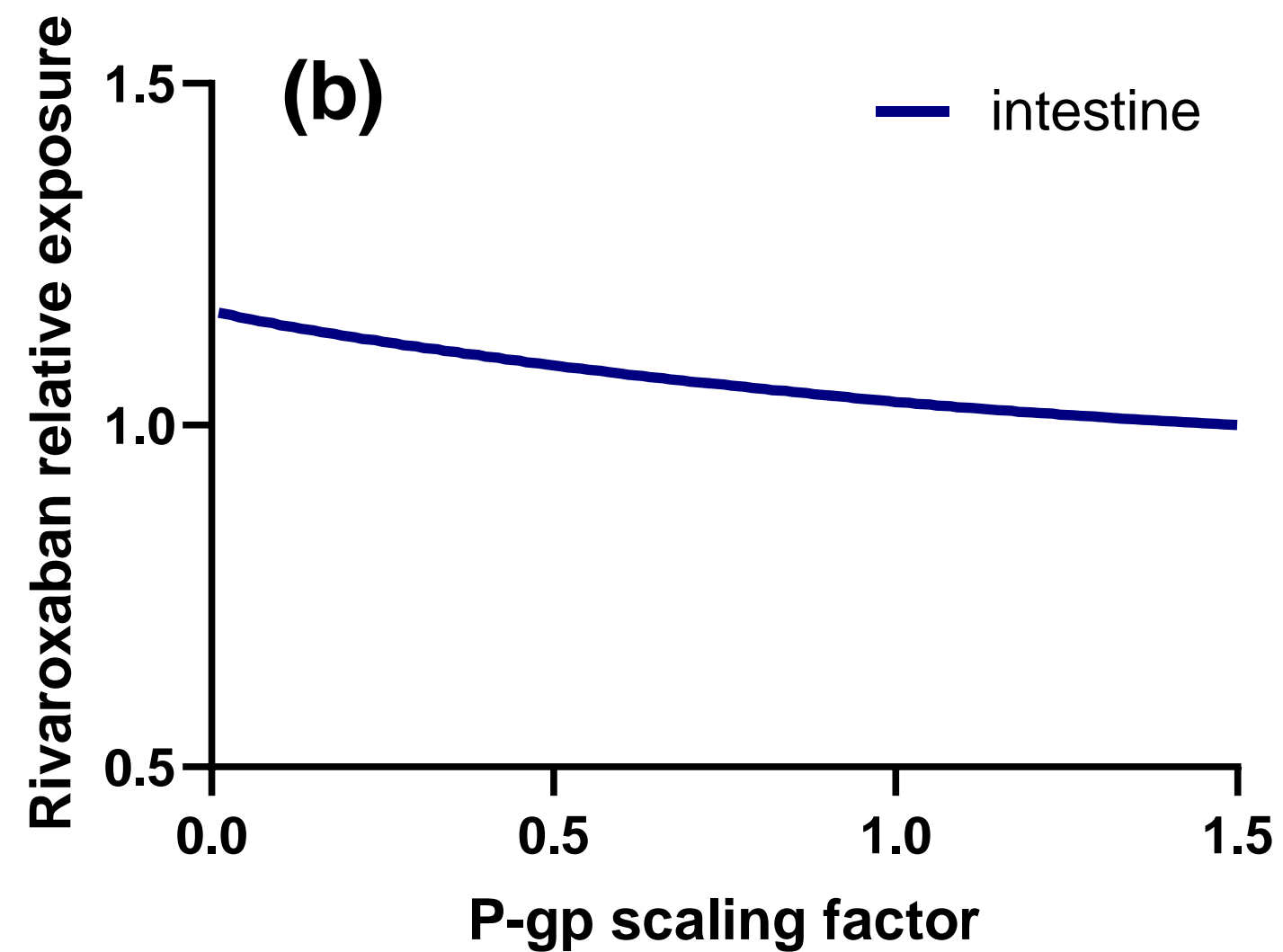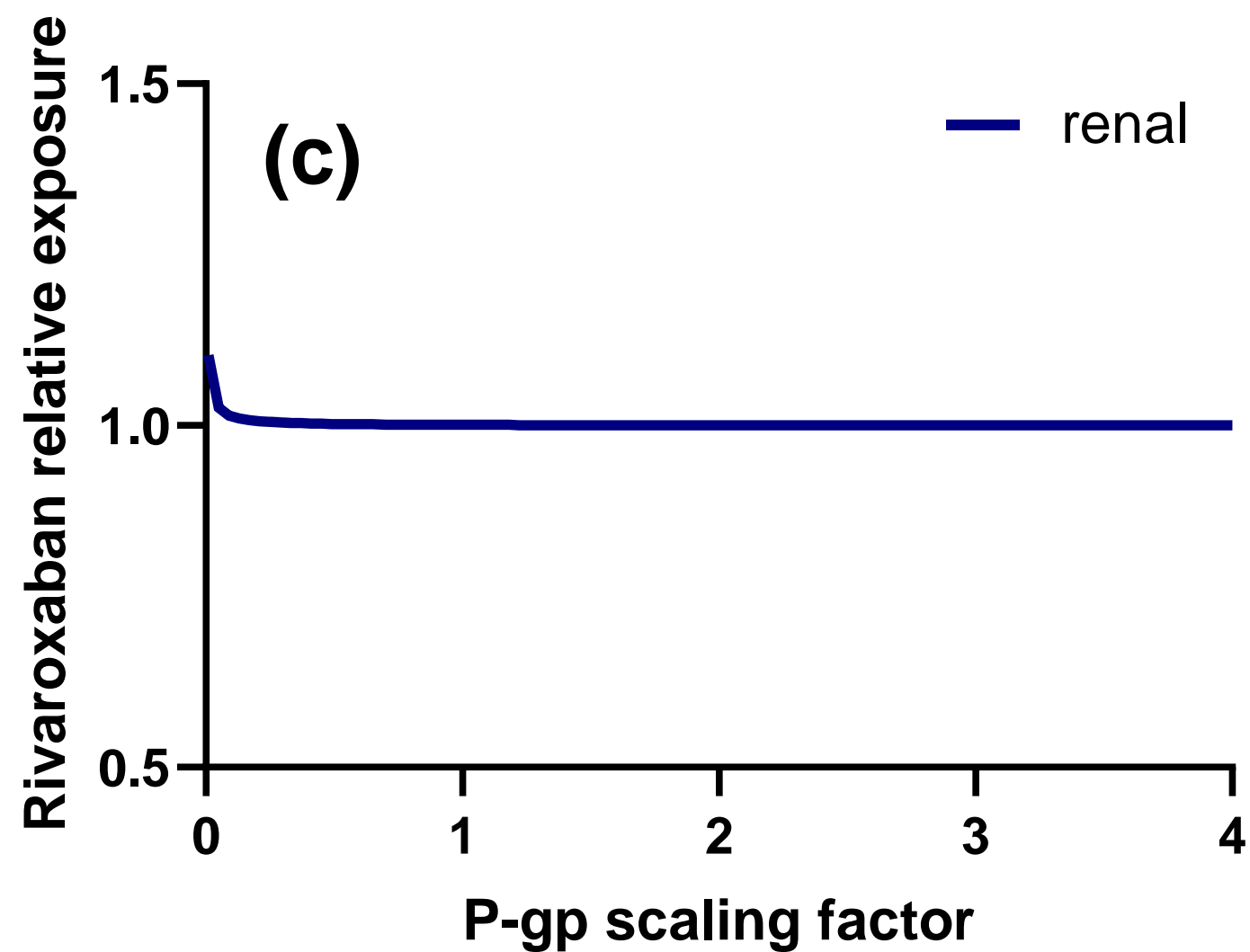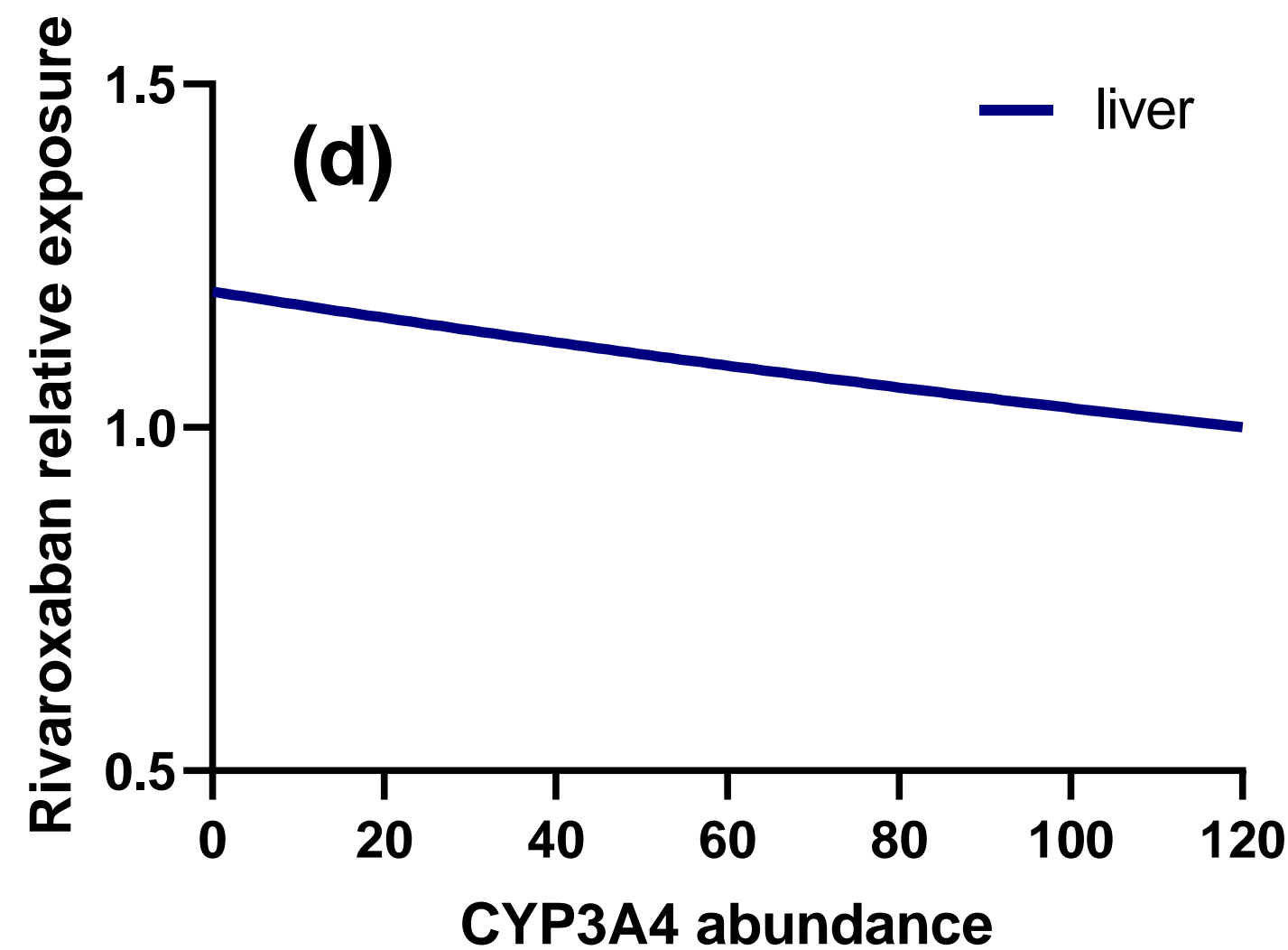

Supplement: Supplementary file 4 [file mmc4.pdf]
