# Supplementary material for: Physiologically-based pharmacokinetic modeling to predict drug-drug interactions of dabigatran etexilate and rivaroxaban in the Chinese older adults
Source: Eur J Pharm Sci. 2023 Mar 1;182:106376. doi: 10.1016/j.ejps.2023.106376 (PMC9883662; doi:10.1016/j.ejps.2023.106376)
Supplement: Supplementary file 2 [file mmc2.pdf]

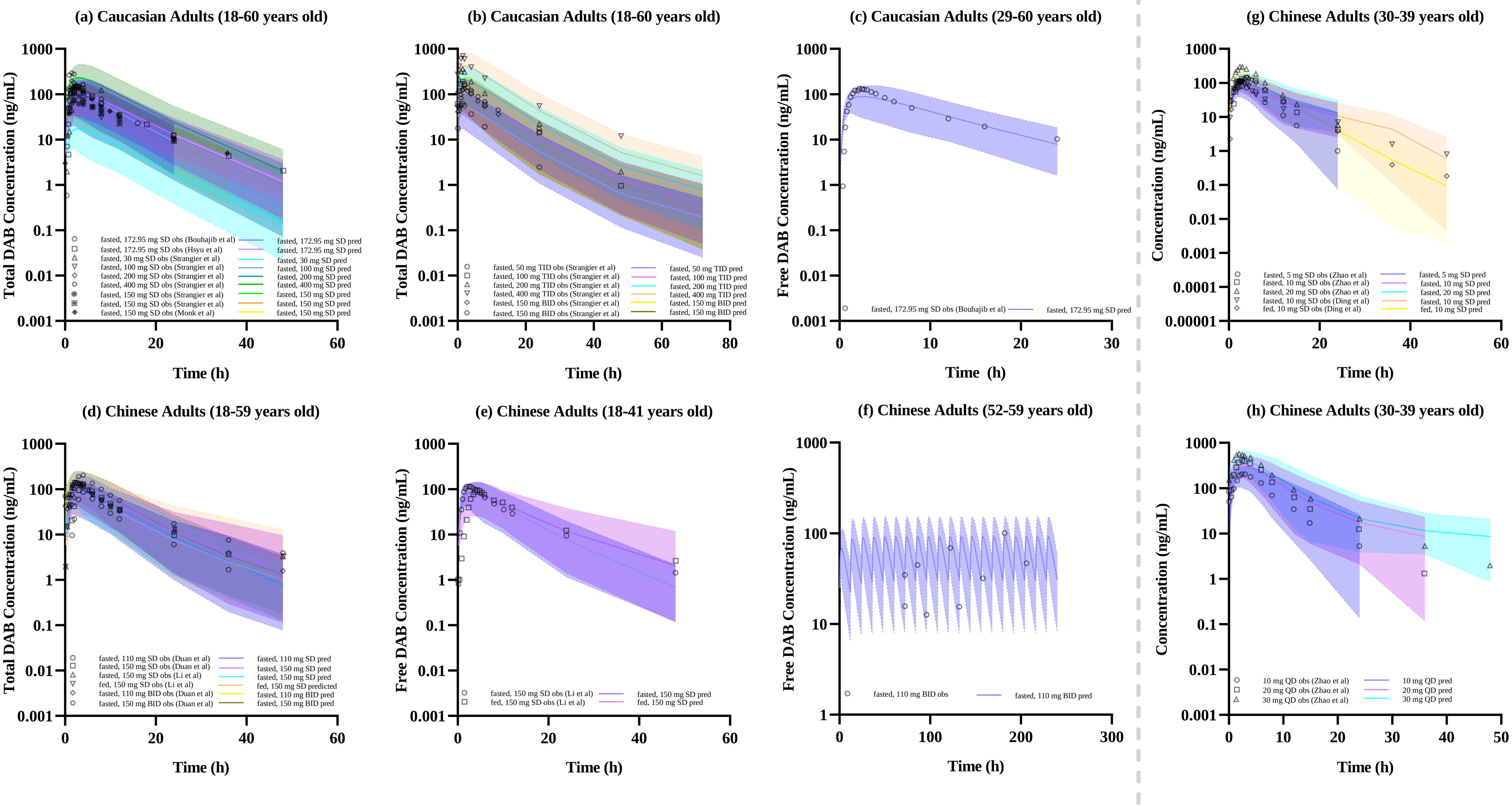
(a) Caucasian Adults (18-60 years old)
Total DAB Concentration (ng/mL)
Time (h)
fasted, 172.95 mg SD obs (Bouhajib et al)
fasted, 172.95 mg SD obs (Hsyu et al)
fasted, 30 mg SD obs (Strangier et al)
fasted, 100 mg SD obs (Strangier et al)
fasted, 200 mg SD obs (Strangier et al)
fasted, 400 mg SD obs (Strangier et al)
fasted, 150 mg SD obs (Strangier et al)
fasted, 150 mg SD obs (Strangier et al)
fasted, 150 mg SD obs (Monk et al)
fasted, 172.95 mg SD pred
fasted, 172.95 mg SD pred
fasted, 30 mg SD pred
fasted, 100 mg SD pred
fasted, 200 mg SD pred
fasted, 400 mg SD pred
fasted, 150 mg SD pred
fasted, 150 mg SD pred
fasted, 150 mg SD pred
(b) Caucasian Adults (18-60 years old)
Total DAB Concentration (ng/mL)
Time (h)
fasted, 50 mg TID obs (Strangier et al)
fasted, 100 mg TID obs (Strangier et al)
fasted, 200 mg TID obs (Strangier et al)
fasted, 400 mg TID obs (Strangier et al)
fasted, 150 mg BID obs (Strangier et al)
fasted, 150 mg BID obs (Strangier et al)
fasted, 50 mg TID pred
fasted, 100 mg TID pred
fasted, 200 mg TID pred
fasted, 400 mg TID pred
fasted, 150 mg BID pred
fasted, 150 mg BID pred
(c) Caucasian Adults (29-60 years old)
Free DAB Concentration (ng/mL)
Time (h)
fasted, 172.95 mg SD obs (Bouhajib et al)
fasted, 172.95 mg SD pred
(g) Chinese Adults (30-39 years old)
Concentration (ng/mL)
Time (h)
fasted, 5 mg SD obs (Zhao et al)
fasted, 10 mg SD obs (Zhao et al)
fasted, 20 mg SD obs (Zhao et al)
fasted, 10 mg SD obs (Ding et al)
fed, 10 mg SD obs (Ding et al)
fasted, 5 mg SD pred
fasted, 10 mg SD pred
fasted, 20 mg SD pred
fasted, 10 mg SD pred
fed, 10 mg SD pred
(d) Chinese Adults (18-59 years old)
Total DAB Concentration (ng/mL)
Time (h)
fasted, 110 mg SD obs (Duan et al)
fasted, 150 mg SD obs (Duan et al)
fasted, 150 mg SD obs (Li et al)
fed, 150 mg SD obs (Li et al)
fasted, 110 mg BID obs (Duan et al)
fasted, 150 mg BID obs (Duan et al)
fasted, 110 mg SD pred
fasted, 150 mg SD pred
fasted, 150 mg SD pred
fed, 150 mg SD predicted
fasted, 110 mg BID pred
fasted, 150 mg BID pred
(e) Chinese Adults (18-41 years old)
Free DAB Concentration (ng/mL)
Time (h)
fasted, 150 mg SD obs (Li et al)
fed, 150 mg SD obs (Li et al)
fasted, 150 mg SD pred
fed, 150 mg SD pred
(f) Chinese Adults (52-59 years old)
Free DAB Concentration (ng/mL)
Time (h)
fasted, 110 mg BID obs
fasted, 110 mg BID pred
(h) Chinese Adults (30-39 years old)
Concentration (ng/mL)
Time (h)
10 mg QD obs (Zhao et al)
20 mg QD obs (Zhao et al)
30 mg QD obs (Zhao et al)
10 mg QD pred
20 mg QD pred
30 mg QD pred
