# Supplementary material for: Physiologically-based pharmacokinetic modeling to predict drug-drug interactions of dabigatran etexilate and rivaroxaban in the Chinese older adults
Source: Eur J Pharm Sci. 2023 Mar 1;182:106376. doi: 10.1016/j.ejps.2023.106376 (PMC9883662; doi:10.1016/j.ejps.2023.106376)
Supplement: Supplementary file 3 [file mmc3.pdf]

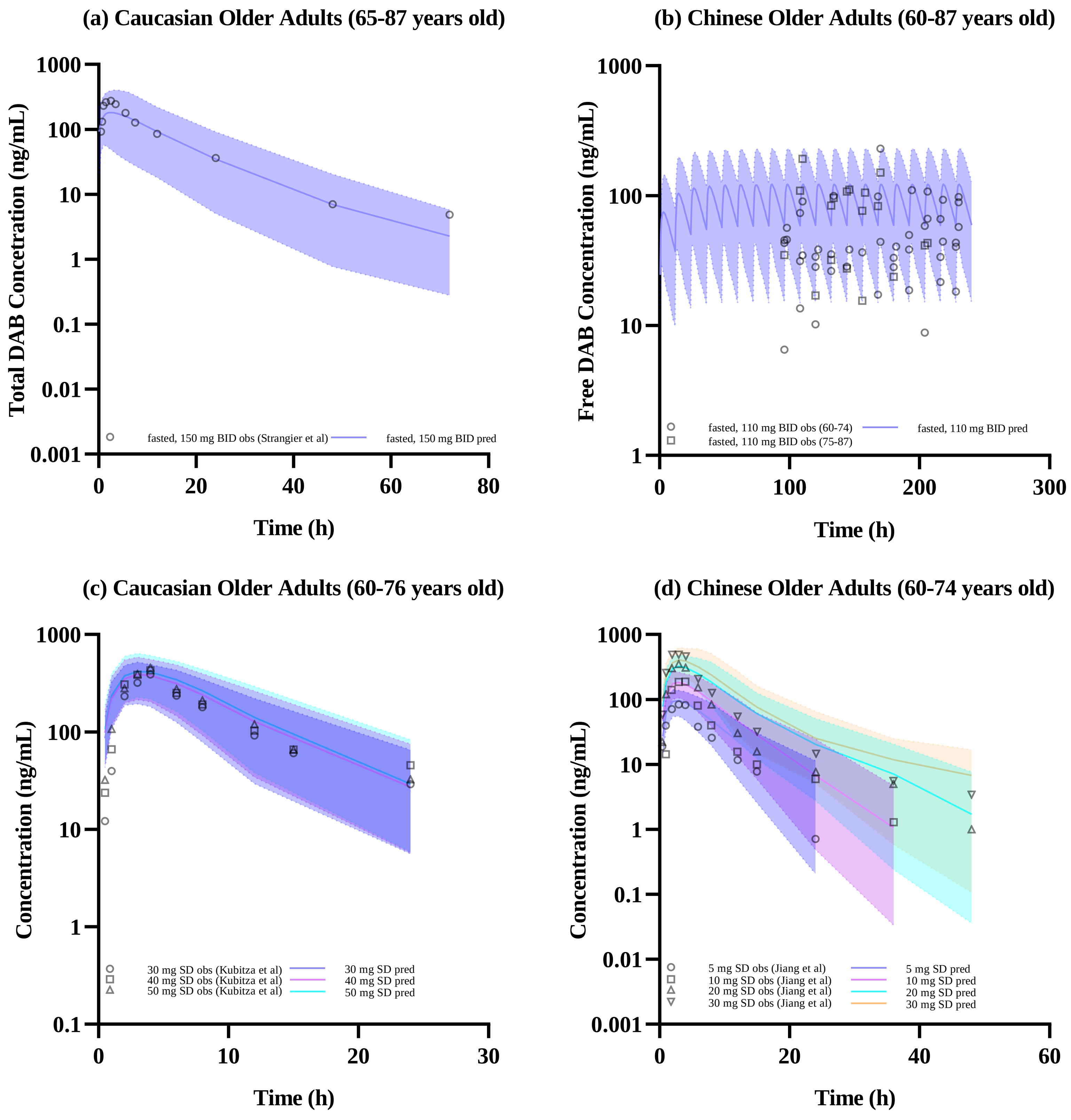

(a) Caucasian Older Adults (65-87 years old)
Total DAB Concetration (ng/mL)
Time (h)
fasted, 150 mg BID obs (Strangier et al)
fasted, 150 mg BID pred
(b) Chinese Older Adults (60-87 years old)
Free DAB Concentration (ng/mL)
Time (h)
fasted, 110 mg BID obs (60-74)
fasted, 110 mg BID obs (75-87)
fasted, 110 mg BID pred
(c) Caucasian Older Adults (60-76 years old)
Concentration (ng/mL)
Time (h)
30 mg SD obs (Kubitza et al)
40 mg SD obs (Kubitza et al)
50 mg SD obs (Kubitza et al)
30 mg SD pred
40 mg SD pred
50 mg SD pred
(d) Chinese Older Adults (60-74 years old)
Concentration (ng/mL)
Time (h)
5 mg SD obs (Jiang et al)
10 mg SD obs (Jiang et al)
20 mg SD obs (Jiang et al)
30 mg SD obs (Jiang et al)
5 mg SD pred
10 mg SD pred
20 mg SD pred
30 mg SD pred
