# Supplementary material for: Physiologically-based pharmacokinetic modeling to predict drug-drug interactions of dabigatran etexilate and rivaroxaban in the Chinese older adults
Source: Eur J Pharm Sci. 2023 Mar 1;182:106376. doi: 10.1016/j.ejps.2023.106376 (PMC9883662; doi:10.1016/j.ejps.2023.106376)
Supplement: Supplementary file 5 [file mmc5.docx]

**Physiologically-Based Pharmacokinetic Modeling to Predict Drug-drug Interactions of Dabigatran Etexilate and Rivaroxaban in the Chinese Older Adults**

Valerie Sia Jie En^1,2,3†^, Xuan Lai^1†^, Xinyi Wu^2,4^, Fan Zhang ^1^, Haiyan Li^2,4^, Cheng Cui^1,2,4#^, Dongyang Liu^2,4,5#^.

^†^Valerie Sia Jie En and Xuan Lai contributed equally to this work.

# Affiliations

^1^Geriatrics Department, Peking University Third Hospital, Beijing 100191, China.

^2^Drug Clinical Trial Center, Peking University Third Hospital, Beijing 100191, China;

^3^Department of Clinical Pharmacy and Pharmacy Administration, School of Pharmacy, Fudan University, Shanghai 201203, China;

^4^Center of Clinical Medical Research, Institute of Medical Innovation and Research, Peking University Third Hospital, Beijing 100191, China.

^5^Beijing Key Laboratory of Cardiovascular Receptors Research, Peking University Third Hospital, Beijing 100191, China.

# #Correspondence

Dongyang Liu

Drug Clinical Trial Center, Peking University Third Hospital, Beijing 100191, China.

Email: liudongyang@vip.sina.com

Cheng Cui

Drug Clinical Trial Center, Peking University Third Hospital, Beijing 100191, China.

Email: cuicheng1226@163.com

**Declaration on Interest:** none

# SUPPLEMENTARY INFORMATION

**Supplementary Text S1: Validation of PBPK model**

The developed models were used and trial designs were set to match population demographics (such as ethnicity, age, female proportion, and blood collection time points) obtained from the 20 literatures (including DDI studies) to simulate the PK characteristics of Caucasian and Chinese adults aged 20-59 years, and Caucasian and Chinese older adults aged ≥ 60 years, given single or multiple doses of dabigatran etexilate (100 mg – 400 mg), or rivaroxaban (2.5 mg – 30 mg) in either fasted or fed state to investigate the predictive performance of the models. For the compound model validation, studies were included when they met the following criteria: (i) The study compound is dabigatran etexilate or rivaroxaban; (ii) PK parameters of the clinical studies were available and/or can be obtained or calculated from the reported plasma and/or whole blood concentration-time profiles; (iii) The clinical studies were conducted in human with subjects (n) ≥ 6; (iv) Chinese or European ancestry aged 18 or above were the only participants in the clinical trials. Also, studies were excluded when (i) the stratification of age in the study population was not defined; (ii) the study population are patients with diseases that will significantly alter the PK of the study compound (i.e, renal impairment, hepatic impairment); (iii) Administration routes other than intravenous infusion (i.v. inf), intravenous bolus (i.v.), or oral; (iv) Extended-release formulation. The dosing regimen used for the model verification were matched to those reported and published in clinical trials as similar as possible. In addition, for clinical studies where dabigatran mesylate was given, 172.95 mg was used as the simulation dose (equivalent to 150 mg dabigatran etexilate) (FDA, 2011). In addition, Otsuka *et al* reported different input parameters used in the contribution of CYP3A4 for hepatic metabolism. A simulation with fluconazole was therefore performed to justify the rationality of the input parameter used in the contribution of CYP3A4 for hepatic metabolism because fluconazole was reported to only inhibits CYP3A4 in the enzymes and transporters involved in rivaroxaban’s disposition.

**LEGENDS**

**Figure S1.** Overall workflow

**Figure S2.** Model verification of DABE and rivaroxaban in Caucasian adults and Chinese adults, after (a, c, d) single dose and (b, e, f) multiple doses of DABE, (g) single dose and (h) multiple doses of rivaroxaban. (f) is presented as steady-state concentration time curve because only individual steady-state concentration data being available. The shaded areas show the 5^th^ and 95^th^ percentile of predicted values. The solid lines represent the mean of predicted value. Black markers represent observed values.

**Figure S3.** Model verification of DABE and rivaroxaban in Caucasian and Chinese older adults, after (a) single dose and (b) multiple doses of DABE, and (c, d) single dose of rivaroxaban. (b) is presented as steady-state concentration time curve because only individual steady-state concentration data being available. The shaded areas show the 5^th^ and 95^th^ percentile of predicted values. The solid lines represent the mean of predicted value. Black markers represent observed values.

**Figure S4.** Sensitivity analysis of (a) intestinal P-gp scaling factor vs free DAB relative exposure, (b) intestinal P-gp scaling factor vs Rivaroxaban relative exposure, (c) renal P-gp scaling factor vs Rivaroxaban relative exposure, and (d) liver CYP3A4 abundance vs Rivaroxaban relative exposure (AUC as exposure metrics).

| Parameters | DABE | Source |
| --- | --- | --- |
| MW (g/mol) | 627.75 | PRADAXA label |
| Log P | 3.8 | (Doki et al., 2019) |
| pKa | 4.0, 6.7 | PRADAXA label |
| Compound type | Diprotic base | PRADAXA label |
| B/P | 1.26 | (Doki et al., 2019) |
| f_u_ | 0.063 | (Doki et al., 2019) |
| **Absorption** |  |  |
| Model | ADAM | (Doki et al., 2019) |
| P_eff,man_ duodenum (10^-4^cm/s) | 0.113 |  |
| P_eff,man_ jejunum I (10^-4^cm/s) | 0.206 |  |
| P_eff,man_ jejunum II (10^-4^cm/s) | 0.144 |  |
| P_eff,man_ ileum I (10^-4^cm/s) | 0.058 |  |
| P_eff,man_ ileum II (10^-4^cm/s) | 0.058 |  |
| P_eff,man_ ileum III (10^-4^cm/s) | 0.057 |  |
| P_eff,man_ ileum IV (10^-4^cm/s) | 0.055 |  |
| P_eff,man_ colon (10^-4^cm/s) | 0.0001 |  |
| **Distribution** |  |  |
| Model | Full PBPK Model |  |
| V_ss_ (L/kg) | 15.16 | Predicted using Method 2 |
| K_p_ scalar | 1.0 |  |
| **Elimination** |  |  |
| HLS9 CES-1 K_m_ (µM) | 33.5 | (Doki et al., 2019) |
| HLS9 CES-1 V_max_ (pmol/min/mg) | 19,462 | (Doki et al., 2019) |
| HLS9 CES-1 tissue scalar (liver/intestine) | 1.0/0 | (Doki et al., 2019) |
| HLS9 CES-2 K_m_ (µM) | 15.4 | (Doki et al., 2019) |
| HLS9 CES-2 V_max_ (pmol/min/mg) | 9,050 | (Doki et al., 2019) |
| HLS9 CES-2 tissue scalar (liver/intestine) | 0.1/0 | (Doki et al., 2019) |
| **Transport (intestinal efflux)** |  |  |
| P-gp K_m_ (µM) | 38.9 | (Doki et al., 2019) |
| P-gp J_max_ (pmol/min/cm^2^) | 146 | (Doki et al., 2019) |

Supplementary Table S1. Input parameters for the development of DABE PBPK model

### Supplementary Table S2. Input parameters for the development of DAB and DAB-G PBPK model

|  | DAB | source | DAB-G | source |
| --- | --- | --- | --- | --- |
| MW (g/mol) | 471.52 | PRADAXA label | 647.65 | (Farhan et al., 2021) |
| Log P | -2.21 | (Doki et al., 2019; Moj et al., 2019) | -4.15 | (Moj et al., 2019) |
| pKa | 4.4, 12.4 | (Doki et al., 2019; Farhan et al., 2021) | 4.14, 3.51 | (Farhan et al., 2021) |
| Compound type | Ampholyte |  | Ampholyte |  |
| B/P | 0.69 | (Doki et al., 2019) | 0.89 | (Farhan et al., 2021) |
| f_u_ | 0.65 | (Doki et al., 2019) | 0.225 | (Farhan et al., 2021) |
| **Distribution** |  |  |  |  |
| Model | Full PBPK |  | Minimal PBPK |  |
| V_ss_ (L/kg) | 0.96 | Predicted using Method 2 | 0.16 | Predicted using Method 2 |
| K_p_ scalar | 3.12 | (Doki et al., 2019) | 1 |  |
| **Elimination** |  |  |  |  |
| UGT2B15 V_max_ (µl/min per picomoles) | 700 | (Farhan et al., 2021) | - |  |
| UGT2B15 K_m_ (µM) | 512 | (Farhan et al., 2021) | - |  |
| CL_r_ (L/h) | 7.97 | (Doki et al., 2019) | 7.97 | Assumed same as DAB renal CL |
| CL­_add_ (L/h) | 0.97 | (Doki et al., 2019) | 0.97 |  |

### Supplementary Table S3. Input parameters for Rivaroxaban PBPK model. (Cheong et al., 2019)

| Parameters | Value |
| --- | --- |
| MW (g/mol) | 435.88 |
| Log P | 1.5 |
| pKa | - |
| Compound type | Neutral |
| B/P | 0.71 |
| f_u_ | 0.065 |
| **Absorption** |  |
| Model | ADAM |
| P_eff,man_ (10^-4^cm/s) | 3.020492 |
| Permeability assay | Caco-2 |
| Apical pH: Basolateral pH | 7.4:7.4 |
| Activity | Passive only |
| P_app,_ A:B (10^-6^cm/s) | 21.8 |
| Reference compound | Multiple |
| Reference compound P_app,_ A:B (10^-6^cm/s) | 0 |
| **Distribution** |  |
| Model | Full PBPK Model |
| V_ss_ (L/kg) | 0.38 |
| V_ss_ Prediction method | Method 2 |
| K_p_ scalar | 2.2 |
| **Elimination** |  |
| CYP3A4 CL_int_ (µl/min per picomoles) | 0.0635 |
| CYP2J2 CL_int_ (µl/min per picomoles) | 5.6854 |
| HLM CL_int_ (µl/min per mg protein) | 7.9988 |
| **Transport (intestinal efflux)** |  |
| Insert growth area of the Transwell (cm^2^) | 0.33 |
| System | MDCK |
| RAF/REF_P-gp_ | 1.5 |
| P-gp K_m_ (µM) | 9.416 |
| P-gp J_max_ (pmol/min) | 37.83 |
| **Transport (kidney)** |  |
| CL_PD,basal_ | 1.09 x 10^-5^ |
| CL_PD,apical_ | 1.09 x 10^-5^ |
| OAT3 CL_int_ | 43 |
| RAF/REF_OAT3_ | 1 |
| P-gp K_m_ (µM) | 9.416 |
| P-gp J_max_ (pmol/min) | 80.921 |
| RAF/REF_P-gp_ | 4 |

### Supplementary Table S4. Input K_i_ value for the use of DDI model

|  | P-gp K_i_  (µM) | CYP3A4 K_i_ (µM) | CYP2J2 K_i_ (µM) | OAT3 K_i_ (µM) | References |
| --- | --- | --- | --- | --- | --- |
| **DABE** |  |  |  |  |  |
| Verapamil | 0.16 | - | - | - | Simcyp embedded value* |
| Clarithromycin | 4.0 | - | - | - | Simcyp embedded value* |
| **Rivaroxaban** |  |  |  |  |  |
| Fluconazole | - | 10.7 | - | - | Simcyp embedded value* |
| Ketoconazole | 0.17 | 0.094 | 0.082 | 0.01 | (Cheong et al., 2019) |
| Clarithromycin | 0.04 | 10.0 | - | - | (Otsuka et al., 2020) |

* Simcyp Simulator (Version 20.1; Certara, Sheffield, United Kingdom) was used.

### Supplementary Table S5. Clinical studies used for model verification of dabigatran etexilate

| Race | Status | Compound of interest |  | Age (min-max or mean, years) | Mean weight (kg) | Dose (mg) | Administration route, dosing schedule | Analytes | Study |
| --- | --- | --- | --- | --- | --- | --- | --- | --- | --- |
| Caucasian | HV | Dabigatran Etexilate | Fed | 29-60 | 79.8 | 150^a^ | Oral, SD | DAB, SUM | (Bouhajib and Tayab, 2020) |
| Caucasian | HV | Dabigatran Etexilate | Fasted | 38.3 | NA | 150 | Oral, SD | SUM | (Härtter et al., 2013) |
| Caucasian | HV | Dabigatran Etexilate | Fasted | 40.1 | NA | 150 | Oral, SD | SUM | (Härtter et al., 2013) |
| Caucasian | HV | Dabigatran Etexilate | Fed | 25-54 | 80.3 | 150^a^ | Oral, SD | SUM | (Hsyu et al., 2017) |
| Caucasian | HV | Dabigatran Etexilate | Fasted | 18-48 | 82.7 | 150 | Oral, SD | SUM | (Monk et al., 2019) |
| Caucasian | HV | Dabigatran Etexilate | Fasted | 18-45 | NA | 100, 200 | Oral, SD | SUM | (Stangier et al., 2007) |
| Caucasian | HV | Dabigatran Etexilate | Fasted | 18-45 | NA | 50, 100, 200, 400 | Oral, MD | SUM | (Stangier et al., 2007) |
| Caucasian | HV | Dabigatran Etexilate | Fasted | 38-66 | 82.25 | 150 | Oral, SD | SUM | (Stangier et al., 2008b) |
| Caucasian | HV | Dabigatran Etexilate | Fasted | 43.4 | 73.0 | 150 | Oral, MD | SUM | (Stangier et al., 2009) |
| Caucasian | HV | Dabigatran Etexilate | Fasted | 20-64 | 74.0 | 150 | Oral, SD | SUM | (Stangier et al., 2010) |
| Caucasian | HV | Dabigatran Etexilate | Fasted | 18-65 | NA | 150 | Oral, MD | SUM | (Stangier et al., 2012) |
| Chinese | HV | Dabigatran Etexilate | Fed | 23-43 | 59.8 | 110 | Oral, SD | SUM | (Duan et al., 2020) |
| Chinese | HV | Dabigatran Etexilate | Fed | 19-37 | 61.2 | 150 | Oral, SD | SUM | (Duan et al., 2020) |
| Chinese | HV | Dabigatran Etexilate | Fasted | 18-39 | 61.5 | 150 | Oral, SD | DAB, SUM | (Li et al., 2020) |
| Chinese | HV | Dabigatran Etexilate | Fed | 18-41 | 51.2 | 150 | Oral, SD | DAB, SUM | (Li et al., 2020) |
| Chinese | P | Dabigatran Etexilate | Fasted | 52-59 | 72.8 | 110 | Oral, MD | DAB | (Zhu et al., 2022) |
| Caucasian | HV | Dabigatran Etexilate | Fasted | 65-87 | 75.1 | 150 | Oral, MD | SUM | (Stangier et al., 2008a) |
| Chinese | P | Dabigatran Etexilate | Fasted | 60-87 | 70.2 | 110 | Oral, MD | DAB | (Zhu et al., 2022) |

HV healthy volunteers, P patients, SD single dose, SUM refers to the total dabigatran concentration (unconjugated dabigatran and free dabigatran), ^a^ Dabigatran Etexilate Mesylate capsule was given

### Supplementary Table S6. Clinical studies used for model verification of rivaroxaban

| Race | Status | Compound of interest |  | Age (min-max, years) | Mean weight (kg) | Dose (mg) | Administration route, dosing schedule | Source |
| --- | --- | --- | --- | --- | --- | --- | --- | --- |
| Chinese | HV | Rivaroxaban | Fasted | 19-35 | 63.0 | 10 | Oral, SD | (Ding et al., 2019) |
| Chinese | HV | Rivaroxaban | Fed | 19-34 | 62.0 | 10 | Oral, SD | (Ding et al., 2019) |
| Chinese | HV | Rivaroxaban | Fasted | 30-39 | 62.4 | 2.5 | Oral, SD | (Zhao et al., 2009) |
| Chinese | HV | Rivaroxaban | Fasted | 30-39 | 66.9 | 5 | Oral, SD | (Zhao et al., 2009) |
| Chinese | HV | Rivaroxaban | Fasted | 30-39 | 58.4 | 10 | Oral, SD | (Zhao et al., 2009) |
| Chinese | HV | Rivaroxaban | Fasted | 32-39 | 59.1 | 20 | Oral, SD | (Zhao et al., 2009) |
| Chinese | HV | Rivaroxaban | Fasted | 32-39 | 62.8 | 40 | Oral, SD | (Zhao et al., 2009) |
| Chinese | HV | Rivaroxaban | Fed | 30-39 | 65.4 | 2.5 | Oral, MD | (Zhao et al., 2009) |
| Chinese | HV | Rivaroxaban | Fed | 31-37 | 64.0 | 5 | Oral, MD | (Zhao et al., 2009) |
| Chinese | HV | Rivaroxaban | Fed | 32-39 | 68.3 | 10 | Oral, MD | (Zhao et al., 2009) |
| Chinese | HV | Rivaroxaban | Fed | 32-39 | 65.6 | 20 | Oral, MD | (Zhao et al., 2009) |
| Caucasian | HV | Rivaroxaban | Fed | 60-76 | 77.3 | 30, 40, 50 | Oral, SD | (Kubitza et al., 2008) |
| Chinese | HV | Rivaroxaban | Fed | 60-65 | 72.5 | 5 | Oral, SD | (Jiang et al., 2010) |
| Chinese | HV | Rivaroxaban | Fed | 60-73 | 59.2 | 10 | Oral, SD | (Jiang et al., 2010) |
| Chinese | HV | Rivaroxaban | Fed | 61-74 | 60.5 | 20 | Oral, SD | (Jiang et al., 2010) |
| Chinese | HV | Rivaroxaban | Fed | 60-72 | 62.2 | 30 | Oral, SD | (Jiang et al., 2010) |
| Chinese | HV | Rivaroxaban | Fed | 60-70 | 63.5 | 40 | Oral, SD | (Jiang et al., 2010) |

HV healthy volunteers, SD single dose, MD multiple dose

### Supplementary Table S7. Summary of simulated and observed values of dabigatran in Caucasian adults and older adults, and Chinese adults and older adults

| Age (min-max or mean, years) | Race | Dosing regimen | AUC_t_ (h·ng/mL) | | | C_max_ (ng/mL) | | | Source |
| --- | --- | --- | --- | --- | --- | --- | --- | --- | --- |
|  |  |  | Obs | Pred | Ratio | Obs | Pred | Ratio |  |
| 29-60 | Caucasian | Fasted, 172.95 mg QD | 1185.4 | 1097.0 | 0.93 | 161.2 | 109.1 | 0.68 | (Bouhajib and Tayab, 2020) |
| 29-60 | Caucasian | Fasted, 172.95 mg QD * | 931.8 | 930.3 | 1.00 | 127.9 | 92.0 | 0.72 | (Bouhajib and Tayab, 2020) |
| 38.3 | Caucasian | Fasted, 150 mg QD | 854.0^a,b^ | 825.7^a,b^ | 0.97 | 99.0^b^ | 83.4^b^ | 0.84 | (Härtter et al., 2013) |
| 40.1 | Caucasian | Fasted, 150 mg QD | 668.0^a,b^ | 825.7^a,b^ | 1.24 | 76.0^b^ | 83.4^b^ | 1.10 | (Härtter et al., 2013) |
| 25-54 | Caucasian | Fed, 172.95 mg QD | 1151.0^b^ | 1096.2^b^ | 0.95 | 129.8^b^ | 88.4^b^ | 0.68 | (Hsyu et al., 2017) |
| 18-48 | Caucasian | Fasted, 150 mg QD | 1030.0^b^ | 859.7^b^ | 0.83 | 128.0^b^ | 80.2^b^ | 0.63 | (Monk et al., 2019) |
| 18-45 | Caucasian | Fasted, 100 mg QD | 548.0 | 696.0 | 1.27 | 82.3 | 60.0 | 0.73 | (Stangier et al., 2007) |
| 18-45 | Caucasian | Fasted, 200 mg QD | 1110.0 | 1395.2 | 1.26 | 161.0 | 120.2 | 0.75 | (Stangier et al., 2007) |
| 18-45 | Caucasian | Fasted, 400 mg QD | 2380.0 | 2803.4 | 1.18 | 344.0 | 241.7 | 0.70 | (Stangier et al., 2007) |
| 18-45 | Caucasian | Fasted, 50 mg TID for 7 days | 305.0^c^ | 353.1^c^ | 1.16 | 42.6^c^ | 53.7^c^ | 1.26 | (Stangier et al., 2007) |
| 18-45 | Caucasian | Fasted, 100 mg TID for 7 days | 904.0^c^ | 707.0^c^ | 0.78 | 128.0^c^ | 107.5^c^ | 0.84 | (Stangier et al., 2007) |
| 18-45 | Caucasian | Fasted, 200 mg TID for 7 days | 1620.0^c^ | 1417.3^c^ | 0.87 | 199.0^c^ | 215.5^c^ | 1.09 | (Stangier et al., 2007) |
| 18-45 | Caucasian | Fasted, 400 mg TID for 7 days | 3270.0^c^ | 2847.9^c^ | 0.87 | 303.0^c^ | 433.2^c^ | 1.43 | (Stangier et al., 2007) |
| 38-66 | Caucasian | Fasted, 150 mg QD | 947.0^a^ | 110.8^a^ | 1.15 | 107.0 | 98.6 | 0.92 | (Stangier et al., 2008b) |
| 43.4 | Caucasian | Fasted, 150 mg BID for 4 days | 1220.0^d^ | 1001.9^d^ | 0.82 | 189.0^d^ | 126.8 | 0.67 | (Stangier et al., 2009) |
| 20-64 | Caucasian | Fasted, 150 mg QD | 901.0^a^ | 1039.0^a^ | 1.15 | 85.3 | 92.1 | 1.10 | (Stangier et al., 2010) |
| 18-65 | Caucasian | Fasted, 150 mg BID for 4 days | 1120.0^c^ | 1001.9^c^ | 0.90 | 167.0^c^ | 126.8^c^ | 0.76 | (Stangier et al., 2012) |
| 23-43 | Chinese | Fed, 110 mg QD | 697.0^a^ | 773.7^a^ | 1.11 | 87.6^b^ | 71.2^b^ | 0.81 | (Duan et al., 2020) |
| 19-37 | Chinese | Fed, 150 mg QD | 1070.0^a^ | 1067.3^a^ | 1.00 | 132.0^b^ | 95.5^b^ | 0.72 | (Duan et al., 2020) |
| 23-43 | Chinese | Fed, 110 mg BID | 805.0^b^ | 994.7^b^ | 1.24 | 133.0^b^ | 96.1^b^ | 0.72 | (Duan et al., 2020) |
| 19-37 | Chinese | Fed, 150 mg BID for 5 days | 1250.0^b^ | 1324.7^b^ | 1.06 | 195.0^b^ | 129.3^b^ | 0.66 | (Duan et al., 2020) |
| 18-41 | Chinese | Fed, 150 mg QD | 1176.1 | 1080.4 | 0.92 | 127.6 | 89.6 | 0.71 | (Li et al., 2020) |
| 18-41 | Chinese | Fed, 150 mg QD * | 1080.9 | 979.3 | 0.91 | 120.7 | 81.2 | 0.67 | (Li et al., 2020) |
| 18-39 | Chinese | Fasted, 150 mg QD | 1227.9 | 1146.1 | 0.93 | 144.9 | 107.4 | 0.74 | (Li et al., 2020) |
| 18-39 | Chinese | Fasted, 150 mg QD * | 1041.6 | 984.6 | 0.94 | 121.9 | 91.2 | 0.75 | (Li et al., 2020) |
| 52-59 | Chinese | Fasted, 110 mg BID for 14 days* | Individual steady-state concentrations ^d^ | | | | | | (Zhu et al., 2022) |
| 65-87 | Caucasian | Fasted, 150 mg BID for 7 days | 1800.0^c^ | 1713.4^c^ | 0.95 | 256.0^c^ | 187.8^c^ | 0.73 | (Stangier et al., 2008a) |
| 60-87 | Chinese | Fasted, 110 mg BID for 14 days | Individual steady-state concentrations ^d^ | | | | | | (Zhu et al., 2022) |

QD once daily, BID twice daily, MD multiple dose, AUC_t_ area under the curve at time 0 to t, C_max_ maximum concentration, ^a^AUC_inf_  (h·ng/mL) area under the curve at time 0 to inf, ^b^AUC and C_­max_ data presented as geometric mean, unlabeled data are presented as arithmetic mean, ^c^AUC_ss_ (h·ng/mL) area under the curve at steady-state, ^d^ only individual steady-state concentrations are available, *only DAB concentration was measured.

### Supplementary Table S8. Summary of simulation and observed values of rivaroxaban in Chinese adults, and Caucasian and Chinese older adults

| Age (years) | Race | Dosing regimen | AUC_t_ (h·ng/mL) | | | C_max_ (ng/mL) | | | Source |
| --- | --- | --- | --- | --- | --- | --- | --- | --- | --- |
|  |  |  | Obs | Pred | Ratio | Obs | Pred | Ratio |  |
| 18-35 | Chinese | Fasted, 10 mg QD | 834.0^a,b^ | 984.5^a,b^ | 1.18 | 127.0 | 105.1 | 0.83 | (Ding et al., 2019) |
| 18-33 | Chinese | Fasted, 10 mg QD | 1098.0^a,b^ | 1245.6^a,b^ | 1.13 | 178.0 | 168.7 | 0.95 | (Ding et al., 2019) |
| 30-39 | Chinese | Fasted, 2.5 mg QD | 251.7^b^ | 303.0^b^ | 1.20 | 51.3^b^ | 40.7^b^ | 0.79 | (Zhao et al., 2009) |
| 30-39 | Chinese | Fasted, 5 mg QD | 410.6^b^ | 580.8^b^ | 1.41 | 67.2^b^ | 67.5^b^ | 1.00 | (Zhao et al., 2009) |
| 30-39 | Chinese | Fasted, 10 mg QD | 1022.0^b^ | 959.5^b^ | 0.94 | 143.2^b^ | 98.0^b^ | 0.68 | (Zhao et al., 2009) |
| 32-39 | Chinese | Fasted, 20 mg QD | 1354.0^b^ | 1240.2^b^ | 0.92 | 204.4^b^ | 126.3^b^ | 0.62 | (Zhao et al., 2009) |
| 32-39 | Chinese | Fasted, 40 mg QD | 1402.0^b^ | 1454.8^b^ | 1.04 | 176.1^b^ | 148.9^b^ | 0.85 | (Zhao et al., 2009) |
| 30-39 | Chinese | Fed, 5 mg BID MD for 6 days | 674.0^b^ | 683.9^b^ | 1.01 | 115.4^b^ | 93.6^b^ | 0.81 | (Zhao et al., 2009) |
| 31-37 | Chinese | Fed, 10 mg BID MD for 6 days | 1305.0^b^ | 1463.9^b^ | 1.12 | 215.9^b^ | 185.3^b^ | 0.86 | (Zhao et al., 2009) |
| 32-39 | Chinese | Fed, 20 mg BID MD for 6 days | 2527.0^b^ | 2660.0^b^ | 1.05 | 415.1^b^ | 309.0^b^ | 0.74 | (Zhao et al., 2009) |
| 32-39 | Chinese | Fed, 30 mg BID MD for 6 days | 3601.0^b^ | 3380.8^b^ | 0.94 | 590.3^b^ | 384.6^b^ | 0.65 | (Zhao et al., 2009) |
| 60-76 | Caucasian | Fed, 30 mg QD | 3531.0^b^ | 3486.2^b^ | 0.99 | 392.0 | 339.0 | 0.86 | (Kubitza et al., 2008) |
| 60-76 | Caucasian | Fed, 40 mg QD | 4385.0^b^ | 4035.8^b^ | 0.92 | 461.0 | 381.3 | 0.83 | (Kubitza et al., 2008) |
| 60-76 | Caucasian | Fed, 50 mg QD | 4496.0^b^ | 4406.9^b^ | 0.98 | 437 | 407.8 | 0.93 | (Kubitza et al., 2008) |
| 60-65 | Chinese | Fed, 5 mg QD | 610.1^a,b^ | 835.1^a,b^ | 1.37 | 121.3 | 98.58 | 0.81 | (Jiang et al., 2010) |
| 60-73 | Chinese | Fed, 10 mg QD | 1060.0^a,b^ | 1649.4^a,b^ | 1.56 | 228.0 | 187.6 | 0.82 | (Jiang et al., 2010) |
| 61-74 | Chinese | Fed, 20 mg QD | 2167.0^a,b^ | 3254.3^a,b^ | 1.50 | 386.2 | 331.6 | 0.86 | (Jiang et al., 2010) |
| 60-72 | Chinese | Fed, 30 mg QD | 3360.0^a,b^ | 4225.3^a,b^ | 1.26 | 550.2 | 417.2 | 0.76 | (Jiang et al., 2010) |

QD once daily, BID twice daily, MD multiple dose, AUC_t_ Area under the curve at time 0 to t, C_max_ maximum concentration, ^a^ AUC_inf_  (h·ng/mL), ^b^ AUC and C_­max_ data presented as geometric mean, unlabeled data are presented as arithmetic mean.

Supplementary Table S9. Observed and simulated exposure change using PBPK-DDI models in Caucasian adults

| Victim | Perpetrator | Dosing regimen |  | Alone | | Concomitantly | | DDI Ratio | |
| --- | --- | --- | --- | --- | --- | --- | --- | --- | --- |
|  |  |  |  | AUC_inf_ (ng·h/mL) | C_max_ (ng/mL) | AUC_inf_ (ng·h/mL) | C_max_ (ng/mL) | AUC | C_max_ |
| Dabigatran Etexilate | Verapamil^a^ | I: 120 mg BID for 1 day, 150 mg DABE given 2 hours before verapamil dose at the same day | Obs | 854.0 | 99.0 | 1010.0 | 111.0 | 1.18 | 1.12 |
|  |  |  | Pred | 825.6 | 83.4 | 1063.7 | 102.9 | 1.29 | 1.23 |
|  |  |  | Ratio / Criteria | 0.97 | 0.84 | 1.05 | 0.93 | 0.87 – 1.62 | 0.84 – 1.49 |
|  | Verapamil^a^ | II: 120 mg QD concomitantly with 150 mg DABE | Obs | 854.0 | 99.0 | 1390.0 | 173.0 | 1.63 | 1.74 |
|  |  |  | Pred | 825.6 | 83.4 | 1439.8 | 139.3 | 1.74 | 1.67 |
|  |  |  | Ratio / Criteria | 0.97 | 0.84 | 1.04 | 0.81 | 1.06 – 2.51 | 1.11 – 2.73 |
|  | Clarithromycin | 500 mg BID for 3 days, and concomitantly with 300 mg DABE at day 4 | Obs | 1507.6 | 182.7 | 1978.5 | 248.0 | 1.31 | 1.36 |
|  |  |  | Pred | 1709.8 | 170.7 | 2184.5 | 219.6 | 1.28 | 1.29 |
|  |  |  | Ratio / Criteria | 1.13 | 0.93 | 1.10 | 0.89 | 0.92 – 1.87 | 0.94 – 1.97 |
| Rivaroxaban | Clarithromycin | 500 mg BID for 5 days, 10 mg rivaroxaban given concomitantly at day 5 under fed condition. | Obs | 964.0 | 139.4 | 1469.0 | 194.4 | 1.52 | 1.39 |
|  |  |  | Pred | 1161.5 | 148.2 | 1503.1 | 188.1 | 1.28 | 1.27 |
|  |  |  | Ratio / Criteria | 1.22 | 1.06 | 1.02 | 0.97 | 1.01 – 2.30 | 0.95 – 2.03 |
|  | Fluconazole | 400 mg QD for 5 days, 20 mg rivaroxaban given concomitantly at day 5 under fed condition. | Obs | 1771.0 | 212.4 | 2464.0 | 268.8 | 1.39 | 1.27 |
|  |  |  | Pred | 2098.5 | 246.9 | 2649.2 | 274.0 | 1.26 | 1.10 |
|  |  |  | Ratio / Criteria | 1.18 | 1.16 | 1.08 | 1.02 | 0.95 – 2.03 | 0.90 – 1.78 |
|  | Ketoconazole | 400 mg QD for 5 days (day5-10), 10 mg rivaroxaban for 10 days (day 1-10) under fed condition. | Obs | 892.0 | 138.1 | 2298.0 | 237.0 | 2.58 | 1.72 |
|  |  |  | Pred | 1158.5 | 145.5 | 3096.5 | 255.6 | 2.67 | 1.76 |
|  |  |  | Ratio / Criteria | 1.30 | 1.05 | 1.35 | 1.08 | 1.51 – 4.41 | 1.10 – 2.69 |

AUC_inf_ area under the curve at time 0 to ꝏ, C_max_ maximum plasma concentration, AUC_inf_ and C_max_ are presented as arithmetic means. ^a^ AUC_inf_ and C_max_ of verapamil is presented as geometric means.

# REFERENCES

Bouhajib, M., Tayab, Z., 2020. A Pharmacokinetic Evaluation of Dabigatran Etexilate, Total Dabigatran, and Unconjugated Dabigatran Following the Administration of Dabigatran Etexilate Mesylate Capsules in Healthy Male and Female Subjects. Drug Res (Stuttg) 70, 33-40.

Cheong, E.J.Y., Teo, D.W.X., Chua, D.X.Y., Chan, E.C.Y., 2019. Systematic Development and Verification of A Physiologically-Based Pharmacokinetic Model of Rivaroxaban. Drug Metabolism and Disposition, dmd.119.086918.

Ding, S., Wang, L., Xie, L., Shao, F., Chen, J., Zhao, Y., Deng, W., Liu, Y., Zhang, H., 2019. Bioequivalence Study of 2 Formulations of Rivaroxaban, a Narrow‐Therapeutic‐Index Drug, in Healthy Chinese Subjects Under Fasting and Fed Conditions. Clinical Pharmacology in Drug Development 9.

Doki, K., Neuhoff, S., Rostami-Hodjegan, A., Homma, M., 2019. Assessing Potential Drug–Drug Interactions Between Dabigatran Etexilate and a P-Glycoprotein Inhibitor in Renal Impairment Populations Using Physiologically Based Pharmacokinetic Modeling. CPT: Pharmacometrics & Systems Pharmacology 8, 118-126.

Duan, J., Yang, L., Li, H., Yamamura, N., Harada, A., 2020. Pharmacokinetics and Safety of Dabigatran Etexilate after Single and Multiple Oral Doses in Healthy Chinese Subjects. European journal of drug metabolism and pharmacokinetics 45, 601-609.

Farhan, N., Cristofoletti, R., Basu, S., Kim, S., Lingineni, K., Jiang, S., Brown, J.D., Fang, L., Lesko, L.J., Schmidt, S., 2021. Physiologically Based Pharmacokinetics Modeling to Investigate Formulation Factors Influencing the Generic Substitution of Dabigatran Etexilate. CPT: Pharmacometrics & Systems Pharmacology.

FDA, U.S., 2011. PRADAXA. Highlights of prescribing information.

Härtter, S., Sennewald, R., Nehmiz, G., Reilly, P., 2013. Oral bioavailability of dabigatran etexilate (Pradaxa(®) ) after co-medication with verapamil in healthy subjects. Br J Clin Pharmacol 75, 1053-1062.

Hsyu, P.H., Pignataro, D.S., Matschke, K., 2017. Effect of bosutinib on the absorption of dabigatran etexilate mesylate, a P-glycoprotein substrate, in healthy subjects. Eur J Clin Pharmacol 73, 57-63.

Jiang, J., Hu, Y., Zhang, J., Yang, J., Mueck, W., Kubitza, D., Bauer, R.J., Meng, L., Hu, P., 2010. Safety, pharmacokinetics and pharmacodynamics of single doses of rivaroxaban - an oral, direct factor Xa inhibitor - in elderly Chinese subjects. Thromb Haemost 103, 234-241.

Kubitza, D., Becka, M., Roth, A., Mueck, W., 2008. Dose-escalation study of the pharmacokinetics and pharmacodynamics of rivaroxaban in healthy elderly subjects. Curr Med Res Opin 24, 2757-2765.

Li, X., Liu, L., Xu, B., Xiang, Q., Li, Y., Zhang, P., Wang, Y., Xie, Q., Mao, Y., Cui, Y., 2020. Bioequivalence and pharmacodynamics of a generic dabigatran etexilate capsule in healthy Chinese subjects under fasting and fed conditions. Pharmacology research & perspectives 8, e00593.

Moj, D., Maas, H., Schaeftlein, A., Hanke, N., Gómez-Mantilla, J.D., Lehr, T., 2019. A comprehensive whole-body physiologically based pharmacokinetic model of dabigatran etexilate, dabigatran and dabigatran glucuronide in healthy adults and renally impaired patients. Clinical pharmacokinetics 58, 1577-1593.

Monk, S.A., Kugler, A.R., Andersen, S.W., Ayan-Oshodi, M.A., James, D.E., Mullen, J., Zimmer, J.A., Willis, B.A., 2019. Clinically Negligible Pharmacokinetic and Pharmacodynamic Interactions Between Lanabecestat and Dabigatran Etexilate, a Prototypical P-gp Substrate. J Clin Pharmacol.

Stangier, J., Rathgen, K., Stähle, H., Gansser, D., Roth, W., 2007. The pharmacokinetics, pharmacodynamics and tolerability of dabigatran etexilate, a new oral direct thrombin inhibitor, in healthy male subjects. Br J Clin Pharmacol 64, 292-303.

Stangier, J., Rathgen, K., Stähle, H., Mazur, D., 2010. Influence of renal impairment on the pharmacokinetics and pharmacodynamics of oral dabigatran etexilate: an open-label, parallel-group, single-centre study. Clin Pharmacokinet 49, 259-268.

Stangier, J., Rathgen, K., Stähle, H., Reseski, K., Körnicke, T., Roth, W., 2009. Coadministration of dabigatran etexilate and atorvastatin: assessment of potential impact on pharmacokinetics and pharmacodynamics. Am J Cardiovasc Drugs 9, 59-68.

Stangier, J., Stähle, H., Rathgen, K., Fuhr, R., 2008a. Pharmacokinetics and pharmacodynamics of the direct oral thrombin inhibitor dabigatran in healthy elderly subjects. Clin Pharmacokinet 47, 47-59.

Stangier, J., Stähle, H., Rathgen, K., Roth, W., Reseski, K., Körnicke, T., 2012. Pharmacokinetics and pharmacodynamics of dabigatran etexilate, an oral direct thrombin inhibitor, with coadministration of digoxin. The Journal of Clinical Pharmacology 52, 243-250.

Stangier, J., Stähle, H., Rathgen, K., Roth, W., Shakeri-Nejad, K., 2008b. Pharmacokinetics and pharmacodynamics of dabigatran etexilate, an oral direct thrombin inhibitor, are not affected by moderate hepatic impairment. J Clin Pharmacol 48, 1411-1419.

Zhao, X., Sun, P., Zhou, Y., Liu, Y., Zhang, H., Mueck, W., Kubitza, D., Bauer, R.J., Zhang, H., Cui, Y., 2009. Safety, pharmacokinetics and pharmacodynamics of single/multiple doses of the oral, direct Factor Xa inhibitor rivaroxaban in healthy Chinese subjects. British journal of clinical pharmacology 68, 77-88.

Zhu, Z., Shen, Z., Shi, A., Su, C., Mao, J., Tao, H., Xu, F., Hu, Z., Pan, J., 2022. Dabigatran plasma concentration indicated the risk of patients with non-valvular atrial fibrillation. Heart and Vessels 37, 821-827.
